# Peptide‐Incorporated Biomaterials Promote Regeneration of Peripheral Nerve Injuries

**DOI:** 10.1002/advs.202524264

**Published:** 2026-03-25

**Authors:** Zhiwei Zhao, Jianfeng Liu, Jincheng Li, Di Li, Jianxun Ding, Bin Liu

**Affiliations:** ^1^ Department of Hand and Podiatric Surgery Orthopedic Center First Hospital of Jilin University Changchun P. R. China; ^2^ State Key Laboratory of Polymer Science and Technology Changchun Institute of Applied Chemistry Chinese Academy of Sciences Changchun P. R. China; ^3^ Engineering Laboratory of Tissue Engineering Biomaterials of Jilin Province Changchun P. R. China; ^4^ Department of Spine Surgery Orthopedic Center First Hospital of Jilin University Changchun P. R. China

**Keywords:** bioactive peptide, biomaterial, peripheral nerve injury, peripheral nerve regeneration, tissue engineering

## Abstract

Peripheral nerve injury (PNI), commonly caused by various forms of trauma, may lead to numbness, muscle weakness, and even loss of motor and sensory function. Autologous nerve transplantation remains the clinical “gold standard” for repairing peripheral nerve defects. However, it has inherent limitations, including limited availability of donor nerves, donor‐site morbidity, and mismatched nerve sizes. Biomaterials‐based nerve guidance conduits offer promising alternatives, particularly when integrated with bioactive factors. Among these, the incorporation of peptides has attracted increasing attention due to its distinct advantages. Incorporated peptides provide precise guidance cues or biological signals to regulate the behaviors of neurons, Schwann cells (SCs), immune cells, and endothelial cells (ECs) after PNI, primarily by mimicking specific functional domains of proteins without the complexity or immunogenicity associated with full‐length proteins. In addition, peptides allow facile structural modification, enabling tunable biological activity, and can be customized and conjugated with high precision to biomaterials. This review summarizes recent progress in peptide‐incorporated biomaterials for facilitating axon elongation, enhancing SC support, modulating inflammatory microenvironments, and inducing vascularization to promote peripheral nerve regeneration, and discusses current challenges and future perspectives for their potential clinical applications.

## Graphical abstract

Peptide‐incorporated biomaterials provide precise, tunable biological cues that mimic functional protein domains to regulate behaviors of neurons, Schwann cells, immune cells, and endothelial cells, thereby enhancing axon elongation, Schwann cell support, inflammatory microenvironment modulation, and vascularization, offering a promising alternative to autologous nerve grafts and advancing peripheral nerve regeneration toward clinical translation.

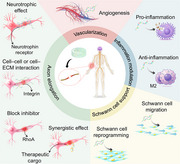

## 1 Introduction

Peripheral nerve injury (PNI), primarily caused by various forms of trauma, such as traffic accidents, sports injuries, and machinery‐related accidents, may compromise motor performance and sensory perception, leading to significant functional decline [1, 2]. Recovery is often poor and does not meet the expectations of clinicians and patients. PNI is a common clinical problem, accounting for approximately 2.6% of upper limb trauma patients, with millions of cases reported annually worldwide [3, 4, 5]. Autologous nerve transplantation remains the “gold standard” for repairing peripheral nerve defects [6]. However, this approach often results in suboptimal functional outcomes [6, 7] and is associated with several limitations, including limited availability of donor nerves, adverse effects at the donor site, and mismatched nerve sizes [5, 8]. These limitations underscore the necessity for advancements in treatment strategies.

Biomaterial‐based nerve guidance conduits (NGCs) have demonstrated clinical potential as alternatives to existing therapeutic methods [9]. They can be artificially synthesized or derived from natural sources and are readily accessible, thereby avoiding donor site damage and associated complications. In addition, they are highly customizable in terms of geometry, mechanical properties, and micro‐ and nano‐architectural features, enabling the fabrication of aligned structures and programmable configurations that better mimic the native nerve microenvironments [10, 11, 12]. They also incorporate bioactive factors, such as peptides, growth factors, cytokines, hormones, and small molecules, to exert synergistic effects that promote nerve regeneration [13, 14]. Among various incorporated bioactive factors, peptides have attracted considerable attention because of their unique advantages. Peptides have molecular weights between those of proteins and small molecules, allowing them to provide precise guidance cues or biological signals that regulate the behaviors of neurons, Schwann cells (SCs), immune cells, and endothelial cells (ECs) after PNI. Compared with proteins, such as many growth factors, peptides generally exhibit superior membrane penetration and lower immunogenicity [15], and they are chemically defined as well as more amenable to structure refinement, such as site‐specific modification using unnatural amino acids or pseudo‐peptide bonds [16, 17]. Compared with small molecules, peptides possess greater chiral and structural complexity, exhibiting higher affinity and specificity toward their targets [15]. Additionally, peptides can be precisely conjugated to biomaterials, generally exhibit good biocompatibility, and can be produced on a large scale. Approximately 80 peptide drugs are already available on the global market, and more than 150 peptides are currently in clinical development [18]. Therefore, peptide‐incorporated biomaterials exhibit significant potential for clinical application in treating PNI.

**SCHEME 1:**
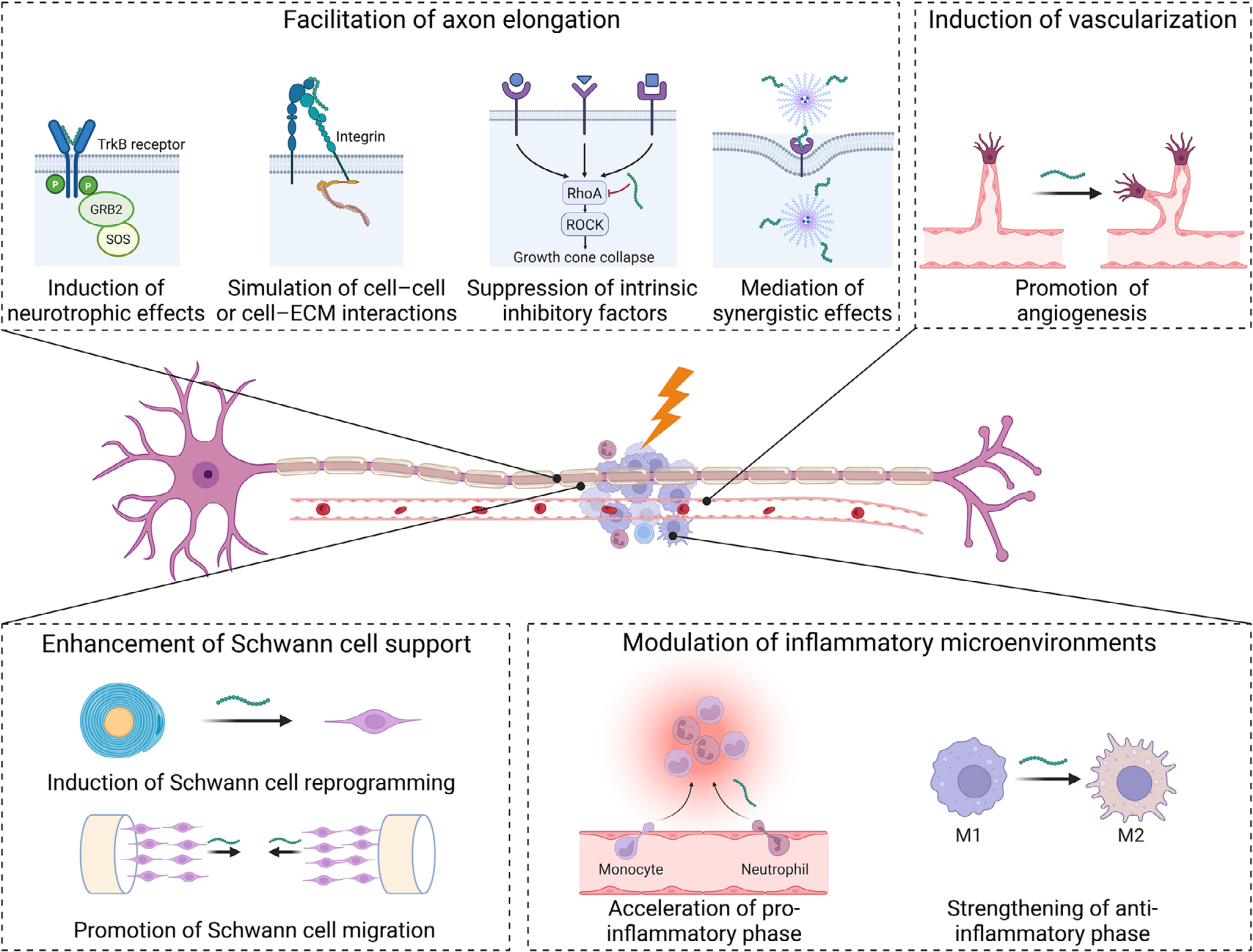
Representative mechanisms by which peptides promote regeneration after PNI can be categorized into several aspects, including facilitation of axon elongation, enhancement of SC support, modulation of inflammatory microenvironments, and induction of vascularization. Created in BioRender. Zhao, Z. (2026) https://BioRender.com/ze8qc66.

Within peptide‐incorporated biomaterial systems, regenerative modulation is significantly influenced by the activities of incorporated peptides. From a mechanistic perspective, peptide incorporation elicits diverse biological activities that contribute to peripheral nerve regeneration (PNR), including facilitation of axon elongation, enhancement of SC support, modulation of inflammatory microenvironments, and induction of vascularization. Each of these four aspects involves specific mechanisms, such as induction of neurotrophic effects, promotion of SC reprogramming and migration, strengthening of the anti‐inflammatory phase, and induction of angiogenesis, as summarized in Scheme 1 and discussed in detail in the following sections.

This review examines the development and recent advances in peptide‐incorporated biomaterials designed to promote PNR and is structured according to the four mechanistic aspects outlined above, with peptides functioning as bioactive factors within these systems. The discussion within each thematic section focuses on cellular and molecular mechanisms that peptide‐based interventions in existing studies have directly targeted. Other biological processes important for PNR but less frequently explored as direct peptide targets are not discussed as standalone sections here. Readers seeking a broader overview of PNR‐related pathophysiology may consult other reviews in this field [19, 20]. The peptides discussed in this review, together with their sequences, derivation, and specific modes of action, are summarized in Table 1. Peptides that primarily function as physical substrates, such as self‐assembling peptides, are not the subject of this article, as they have been thoroughly reviewed elsewhere [21, 22, 23]. The review concludes with a discussion of current challenges and future perspectives regarding their clinical translation, providing insights into the further development of peptide‐incorporated biomaterial‐based strategies for nerve repair.

**TABLE 1:** Names, sequences, sequence origins, and mechanisms of action of bioactive peptides.

Biological activity	Peptide name	Peptide sequence	Source of peptide sequence	Mechanism	References
Induction of neurotrophic effects	RGI peptide	RGIDKRHWNSQ	Derived mainly from a solvent‐exposed loop of BDNF.	Bind to and activate the TrkB receptor, promoting neurite outgrowth and neuronal survival through phosphorylation of intracellular kinases Akt and ERK.	[42]
	IKRG peptide	IKRG	Identified through epitope mapping of neutralizing antibodies to human BDNF	Function as a partial TrkB agonist in a dose‐dependent manner and induce expression of both BDNF and TrkB to establish a positive feedback mechanism.	[50, 51, 52]
	CCK‐8	DY (SO_3_H) MGWMDF	C‐terminal bioactive fragment of cholecystokinin (CCK)	Peripheral administration elevates endogenous NGF protein and mRNA levels in peripheral tissues.	[54, 55, 56]
	Exendin‐4 / Exenatide (synthetic exendin‐4)	HGEGTFTSDLSKQMEEEAVRLFIEWLKNGGPSSGAPPPS	GLP‐1 analogue isolated from Gila monster saliva	Protect neurons by activating GLP‐1 receptors and downstream cAMP‐mediated neuroprotective signaling cascades.	[59, 60, 61, 63]
Simulation of cell−cell interactions	HNK‐1 glycomimetic peptide	FLHTRLFV	Isolated via phage display method for peptide mimics binding specifically to HNK‐1 antibodies.	Attach to CAMs (such as NCAM, L1, MAG, and integrins), mediating Schwann‐cell−motor‐neuron interactions and preferentially promoting motor neurite outgrowth and extension.	[70, 71, 73]
Simulation of cell−ECM interactions	IKVAV motif	IKVAV	Originating from laminin α‐1 chain, identified as an active site responsible for cell adhesion and neurite outgrowth	Interact with the β1‐integrin subunit to enhance neurite outgrowth, neuronal viability, and maturation.	[81, 84, 85]
Suppression of intrinsic inhibitory factors	C3bot 156−181 peptide (C3^156−181^)	SFKLGAVWEKRKSGKLRVLDKVRGI	Derived from *Clostridium botulinum* C3‐exoenzyme (C3bot)	Suppress RhoA/ROCK signaling, reducing growth‐cone collapse and neuronal death, thus promoting axon elongation toward motor targets.	[89, 95]
	G3BP1 B‐domain peptide (190−208)	VV (EP)_7_ VSD	Corresponds to amino acids 190−208 of the G3BP1 B‐domain	Disassemble G3BP1 granules that sequester mRNAs within injured axons, restoring local translation and enhancing axon regeneration.	[99, 100]
Mediation of synergistic effects	Tet‐1 peptide	HLNILSTLWKYR	Derived from tetanus toxin using phage display	Bind to molecules like GT1b gangliosides and sphingophospholipids on neurons, enabling cellular uptake and retrograde axonal transport of therapeutics.	[103, 104, 105]
	RVG29 peptide	YTIWMPENPRPGTPCDIFTNSRGKRASNG	Originates from rabies virus glycoprotein	Specifically bind neuronal nicotinic acetylcholine receptors (nAChRs) to enable neuron‐targeted delivery.	[106, 107]
	DRG homing peptides	SPGARAF (DRG1) DGPWRKM (DRG2) FGQKASS (DRG3)	Identified via in vitro phage display screening for binding affinity to DRG neurons	Selectively recognize and are internalized by DRG neurons to achieve targeted delivery.	[109, 110]
Induction of SC reprogramming	LRP1 agonist peptide 2	SGRGKMLLFSGRRLWRFDVKAQ	Derived from hemopexin domain (PEX) of MMP‐9, a known LRP1 ligand	Bind and activate LRP1, triggering pro‐survival signaling (MAPK/ERK, c‐Jun, and PI3K‐Akt) and enhancing SC repair responses.	[122]
Promotion of SC migration	KHI motif	KHIFSDDSSE	Derived from neural cell adhesion molecule (NCAM)	Induce directional migration of SCs toward KHI gradients while suppressing fibroblast motility.	[69, 125, 126, 127]
	YIGSR motif	YIGSR	Derived from laminin β1 chain	Guide SC migration along YIGSR gradients, showing selective haptotactic responses over fibroblasts.	[124]
	GIP	YAEGTFISDYSIAMDKIHQQDFVNWLLAQKGKKNDWKHNITQ	Endogenous peptide hormone	The GIP/GIPR axis promotes SC migration through PKA/RAP1/mTORC2 signaling.	[130, 131, 132]
Acceleration of pro‐inflammatory phase	TNF‐ mimic peptide (Seq ID n° 2)	Not reported	Selected via phage display technology	Bind to TNF‐α receptors to recruit macrophages and promote the progression of Wallerian degeneration during early nerve repair.	[161, 162]
	NP‐1	ACYCRIPACIAGERRYGTCIYQGRLWAFCC	Endogenous antimicrobial and immunoregulatory peptide	Promote macrophage phagocytosis, proliferation, and migration, accelerating early pro‐inflammatory axonal debris clearance during Wallerian degeneration.	[155]
Strengthening of anti‐inflammatory phase	SP	RPKPQQFFGLM	Endogenous neuropeptide	Act through neurokinin receptors (NKRs), particularly NK1R, to modulate immune responses, particularly M2 macrophage polarization, thereby promoting an anti‐inflammatory microenvironment during PNR.	[137, 138]
	SDF‐1α mimetic peptide	SKPVVLSYR	Retains receptor‐ activating domain of SDF‐1α	Coordinate immune cell migration and activation primarily through CXCR4 and ACKR3, playing a crucial role in inflammation as well as in hematopoiesis and angiogenesis.	[141, 142, 144, 145]
	ARA290	QEQLERALNSS	Derived fromhelix B domain of erythropoietin (EPO)	Selectively activate the tissue protective receptor (TPR) complex without engaging (EPOR)_2_, conferring anti‐inflammatory and tissue‐protective effects without hematopoietic activity.	[147, 148, 149, 150]
	Tkip	WLVFFVIFYFFR	Designed by reading complementary strand of JAK2 autophosphorylation site	Bind to the JAK2 auto‐phosphorylation site, inhibiting its activation and downregulating the JAK‐STAT pathway to suppress pro‐inflammatory cytokines including IFN‐γ, TNF‐α, and IL‐1β.	[151, 153, 154]
Promotion of angiogenesis	QK peptide	KLTWQELYQLKYKGI	Based on 17−25 helix region of VEGF	Bind to and activate VEGFR2, triggering downstream signaling pathways (such as FAK, ERK1/2, and AKT1) that respectively enhance EC migration, proliferation, and permeability.	[115, 169, 173]
	CH02 peptide	GPANVET	Identified via phage display screening for ligands targeting FGFR2.	Bind with high affinity to extracellular domain of FGFR2 to activate FGFR signaling, and also interact with several other RTKs—including VEGFR2—to exert angiogenic effects.	[174, 175]

## 2 Peptides Facilitate Axon Elongation

**SCHEME 2:**
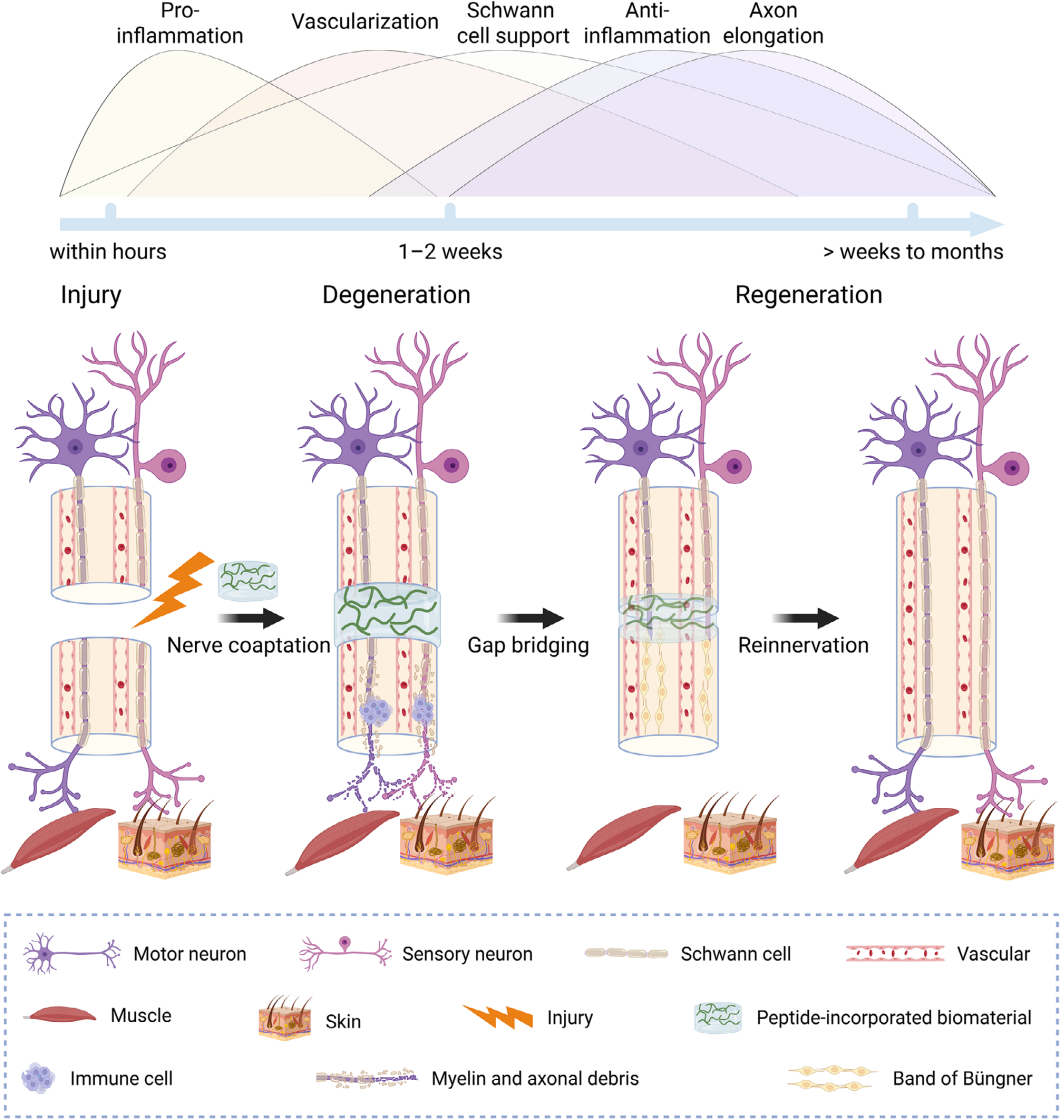
Dynamic sequence of degenerative and regenerative events following PNI and their modulation by peptide‐incorporated biomaterials during nerve coaptation. The early degenerative phase is characterized by Wallerian degeneration, which is associated with pro‐inflammatory responses that prepare the microenvironments for subsequent regeneration [26]. This is followed by a regenerative phase characterized by SC‐mediated structural and trophic support, revascularization, an anti‐inflammatory transition, axon elongation, and subsequent remyelination [27, 28]. Upon implantation, peptide‐incorporated biomaterials modulate specific regenerative processes within this temporally overlapping sequence, thereby facilitating nerve gap bridging, reinnervation, and functional maturation [24, 25]. Created in BioRender. Zhao, Z. (2026) https://BioRender.com/dlnhsfv.

Within the dynamic, multi‐stage repair sequence depicted in Scheme 2, PNR progresses from an initial degenerative phase to subsequent regenerative phases, during which multiple cellular and molecular processes unfold in a broadly sequential manner while also exhibiting temporal overlap [24, 25]. Early Wallerian degeneration, occurring primarily in the distal nerve segment, and the associated pro‐inflammatory response prepare the tissue microenvironments [26], which are subsequently reshaped by SC support, vascular remodeling, and an anti‐inflammatory transition [27, 28]. Against this evolving background, neuronal axon elongation represents a pivotal regenerative process that ultimately determines whether functional connections can be re‐established after PNI with loss of axonal continuity. Axon elongation is the process by which damaged neurons regenerate their axons toward their target tissues, such as muscle or skin [29]. This process is led by the growth cone, an actin‐rich structure at the axonal tip that detects and responds to microenvironmental cues [30]. Regenerating axons advance along the bands of Büngner formed by aligned SCs. Upon reaching their targets, the axons establish synaptic connections or neuromuscular junctions [31, 32]. However, axon elongation after PNI is hindered by various factors, including disruption of retrogradely transported endogenous neurotrophins, insufficient interactions between materials and cells, increased inhibitory factors, and restricted neuronal entry of therapeutic cargos. This section, therefore, outlines peptide‐based strategies that counter these barriers by inducing neurotrophic effects, mimicking cell−cell or cell−extracellular matrix (ECM) interactions, suppressing intrinsic inhibitory factors, or acting synergistically with other therapeutic agents.

### 2.1 Induction of Neurotrophic Effects

The induction of neurotrophic effects is crucial for axon elongation and nerve regeneration. Neurotrophins and their receptors typically mediate neurotrophic effects. Peripheral nerve axotomy disrupts endogenous retrogradely transported neurotrophins, subsequently leading to neuronal cell death and impaired regeneration [33]. Neurotrophins, including nerve growth factor (NGF), brain‐derived neurotrophic factor (BDNF), neurotrophin‐3 (NT‐3), neurotrophin‐4 (NT‐4), and glial cell line‐derived neurotrophic factor (GDNF), exert their functions by binding to neurotrophin receptors, mainly the tropomyosin receptor kinase (Trk) receptors (TrkA, TrkB, and TrkC) and p75^NTR^ [34, 35, 36]. The administration of exogenous neurotrophins following nerve injury has been shown to exert beneficial effects on axon elongation and nerve regeneration [33]. At the molecular level, neurotrophin‐induced axon elongation is primarily mediated through activation of Trk receptors and downstream signaling pathways, particularly the Ras‐Raf‐MEK‐ERK and PI3K‐Akt cascades. These pathways regulate axonal growth through both local modulation of cytoskeletal dynamics at the growth cone and retrograde signaling via internalized neurotrophin−Trk complexes, which activate transcriptional programs, such as cAMP‐responsive element binding protein (CREB)‐dependent gene expression [37]. However, the use of exogenous neurotrophins is constrained by their controversial sources, high cost, short half‐lives, and susceptibility to degradation [38, 39, 40]. Neurotrophin‐mimetic peptides provide a promising strategy to overcome these limitations associated with full‐length neurotrophin proteins [41]. Because the ability of neurotrophins to bind and activate their receptors depends on specific peptide motifs within their molecular chains, researchers have designed synthetic peptides that mimic these functional domains and incorporated them into biomaterials to promote axon elongation and PNR.

**FIGURE 1:**
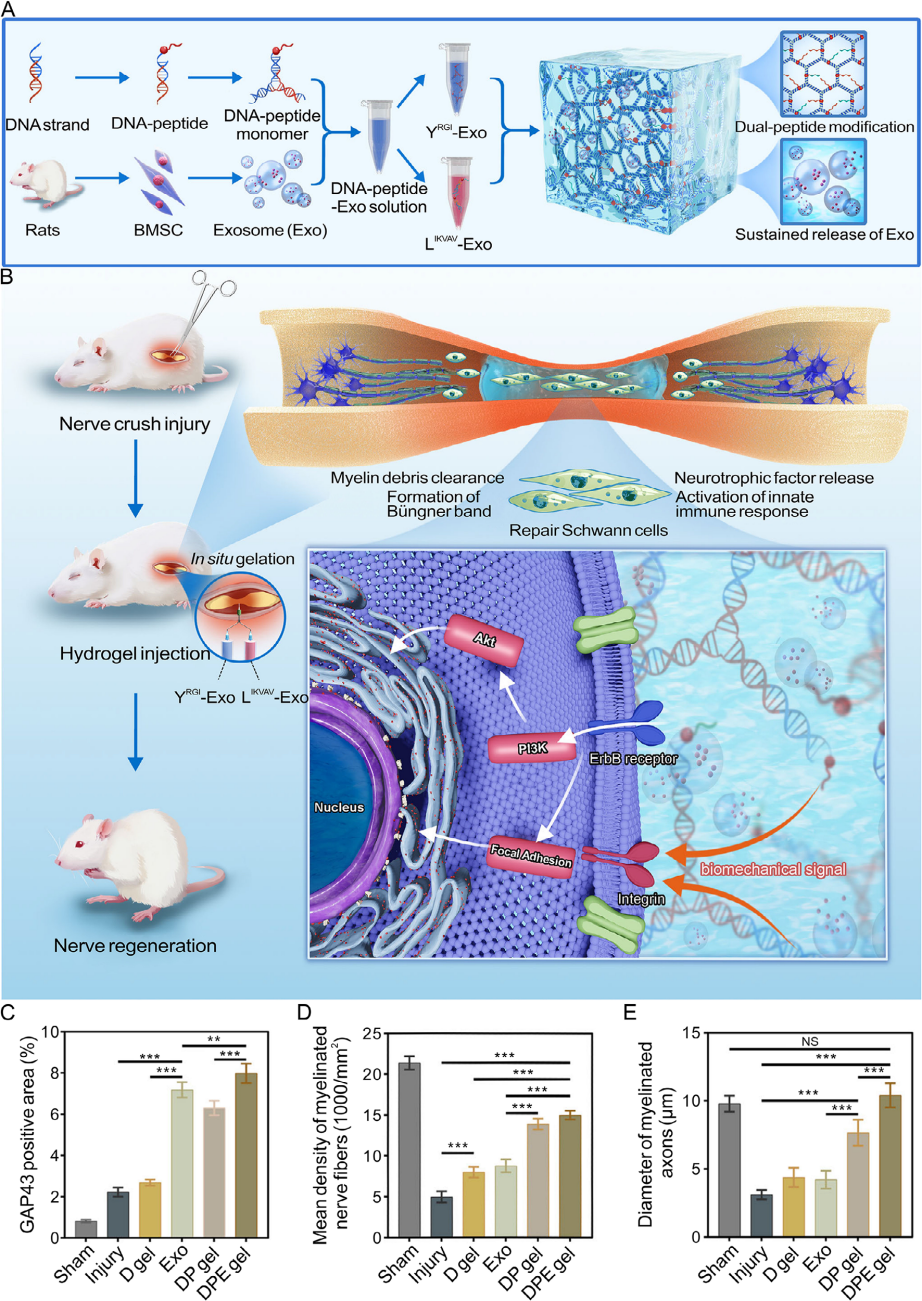
Peptide‐incorporated exosome‐loaded DNA hydrogels for sequential modulation of PNR. (A) Development of programmable DNA‐peptide conjugated, exosome‐loaded (DPE) hydrogel. (B) Formed hydrogel supported the sequential modulation of PNR. (C) GAP43 expression area in regenerated nerves increased significantly after peptide‐incorporated DPE hydrogel treatment three days post‐injury in a rat model of sciatic nerve crush injury. (D) Quantitative analysis of mean density of myelinated nerve fibers based on toluidine blue‐stained images of regenerated sciatic nerves in different experimental groups, 28 days post‐injury. (E) Quantitative analysis of the diameter of myelinated axons. Reproduced with permission [49]. Copyright 2025, Wiley.

The RGI peptide has been extensively investigated as a BDNF‐mimetic peptide for treating PNI. Fobian et al. reported that this peptide induces neurite outgrowth and promotes neuronal survival [42]. The peptide sequence is derived mainly from a solvent‐exposed loop of BDNF and is capable of binding and activating the TrkB receptor, the primary receptor for BDNF [42]. Lu et al. conjugated the RGI peptide to the C‐terminus of a self‐assembling peptide RADA16‐I and prefilled the resultant hydrogel within the lumen of a chitosan conduit. The incorporation of RGI created a neurotrophic microenvironments that significantly enhanced axonal regeneration and motor functional recovery in a rat sciatic nerve defect model [43]. Based on these findings, many studies have combined the RGI peptide with other functional peptides to achieve synergistic effects that promote PNR [38, 44, 45, 46, 47, 48]. For example, the RGI and IKVAV peptides were precisely and stably integrated into DNA monomers through a strain‐promoted azide−alkyne cycloaddition (SPAAC) reaction. The peptide‐incorporated DNA hydrogels were fabricated through DNA sequence self‐assembly and were loaded with bone marrow mesenchymal stem cell‐derived exosomes (Figure 1A). In a rat sciatic nerve crush injury model, intraneural injection of this composite hydrogel enabled in situ gelation, thus facilitating the sequential modulation of PNR (Figure 1B). The GAP43‐positive area in regenerated nerves significantly increased three days post‐injury after treatment with the DNA−peptide conjugated, exosome‐loaded (DPE) hydrogel (Figure 1C). Furthermore, the mean density of myelinated nerve fibers and the diameter of myelinated axons in regenerated sciatic nerves were markedly enhanced 28 days post‐injury (Figure 1D,E) [49].

The IKRG peptide is another BDNF‐mimetic peptide. It was identified by Cardenas‐Aguayo et al. through screening a series of short peptides, mainly tetrapeptides, designed to mimic the function of BDNF while enhancing its permeability and stability [50]. A poly(ethylene glycol)−poly(ε‐caprolactone) (PEG−PCL) nanoparticle that had been surface‐modified with IKRG peptides mimicked the function of BDNF and specifically targeted TrkB receptors to regulate neuronal activity. Increased Akt expression and neurite extension were observed in dorsal root ganglion (DRG) neurons, leading to improved nerve regeneration [51, 52].

Notably, in addition to the peptides mentioned above, many other peptides have been developed to mimic the function of specific neurotrophins. However, they have not yet been utilized in research on PNR [36]. For example, currently developed BDNF‐mimetic peptides include monomeric and dimeric cyclopeptide analogs, linear tetrapeptide analogs, and dimeric dipeptide mimetics. Each exhibits distinct selectivity and pharmacokinetic characteristics [53], and some may offer particular advantages for treating PNI and require further study.

Although some peptides are not neurotrophins and lack direct neurotrophic effects, they may induce endogenous neurotrophin synthesis and thereby exert neurotrophic activity. For example, the neuropeptide cholecystokinin‐8 (CCK‐8), initially recognized as a gastric factor involved in feeding behavior regulation, was later identified as a key signaling molecule in the peripheral and central nervous systems. Exogenous peripheral administration of CCK‐8 enhances endogenous NGF expression at both the mRNA and protein levels in peripheral tissues, thereby facilitating functional recovery in an animal model of sensory deficit. This effect is thought to be mediated by CCK receptor‐dependent intracellular signaling, although the precise downstream pathways remain to be fully elucidated [54, 55, 56].

In addition to classical neurotrophins and their receptors, several other ligand−receptor pairs exhibit neurotrophic effects. For example, the glucagon‐like peptide‐1 receptor (GLP‐1R), which binds GLP‐1, is a G protein‐coupled receptor (GPCR) with a pivotal role in glucose metabolism [57]. In addition to its presence in the pancreas, it is expressed in the nervous system, including DRG neurons and SCs, where it exhibits neurotrophic and neuroprotective properties [58, 59]. Compared with GLP‐1, other GLP‐1R agonists, such as exendin‐4 (Ex‐4) and exenatide, exhibit significantly longer plasma half‐lives because of their resistance to degradation by dipeptidyl peptidase IV [60]. As a natural GLP‐1 analog, Ex‐4 is derived from the saliva of glia monsters [61]. Repeated intraperitoneal injections of Ex‐4 promoted axonal regeneration and facilitated functional recovery following sciatic nerve crush injury in rats [62]. Mechanistically, Takaku et al. established a co‐culture system of rat DRG neurons and SCs IFRS1. Their findings suggest that Ex‐4 activates GLP‐1R‐dependent PI3K‐Akt signaling pathway in both cell types, thereby supporting neurite outgrowth [59]. Given that GLP‐1R is capable of engaging multiple intracellular pathways, including the cAMP‐PKA and MAPK‐ERK cascades, additional signaling mechanisms may also contribute, although their roles in PNR remain to be clarified. Exenatide, the synthetic form of Ex‐4, is currently administered subcutaneously to treat type 2 diabetes and is US Food and Drug Administration (FDA)‐approved [63]. After subcutaneous injection in a rat sciatic nerve transection injury model, exenatide exerted neurotrophic effects, leading to a significant increase in axon number, and improved electrophysiological and motor functions at 12 weeks post‐surgery [64]. While downstream molecular mechanisms were not directly examined in this study, these effects are presumed to involve GLP‐1R–mediated intracellular signaling similar to that of Ex‐4.

### 2.2 Simulation of Cell−Cell or Cell−Extracellular Matrix Interactions

Cell−cell and cell−ECM interactions profoundly regulate neuronal behaviors, including polarization, cytoskeletal organization, and motility, which are particularly critical for axon elongation after PNI [65]. Peptides have been engineered to simulate these interactions and thereby enhance the regenerative capacity of biomaterials. The molecular basis of these interactions primarily involves three protein classes: (i) cell adhesion molecules (CAMs), which are generally transmembrane glycoproteins on the cell surface that couple neighboring cells, via binding to CAMs or other receptors, and ECM proteins to the cytoskeleton, while also serving as signal transducers [65], (ii) ECM proteins, and (iii) cytoplasmic plaque proteins, which are positioned at the intracellular surface of plasma membrane and connect adhesion systems to the cytoskeleton [66]. These interactions are dynamic and driven by the association and dissociation of macromolecular bonds [67].

Cell−cell interactions contribute significantly to axonal elongation. These interactions are predominantly mediated by CAMs, which function through interacting with CAMs or other receptors on adjacent cell surfaces [68]. Given their structural diversity, CAMs are categorized into several families, most notably cadherins, integrins, selectins, and the immunoglobulin‐like CAM (Ig‐CAM) family [65, 69]. Numerous CAMs undergo glycosylation, one of the most abundant post‐translational modifications, which plays a critical role in regulating their biological functions [68]. A notable example of glycosylation‐dependent cell−cell interactions involved in axon elongation is the HNK‐1 carbohydrate, a specialized glycan that regulates interactions between motor neurons and SCs, thereby promoting motor neuron neurite outgrowth [70, 71]. At the molecular level, HNK‐1 can further engage amphoterin (HMGB1) at the cell surface, thereby linking CAM‐mediated recognition to the receptor for advanced glycation end products (RAGE)‐dependent signaling that supports neurite outgrowth, motoneuron survival, and preferential motor reinnervation [72]. In the peripheral nervous system (PNS), the HNK‐1 carbohydrate is present on myelinating SCs associated with motor axons, but not on SCs associated with sensory axons [71]. By attaching to CAMs, such as NCAM, L1, and myelin‐associated glycoprotein (MAG), the HNK‐1 carbohydrate plays a critical role in mediating cell−cell interactions [73]. HNK‐1 carbohydrate‐coated substrates promote neurite outgrowth in motor neurons in vitro while exerting no effect on sensory neurons. However, the therapeutic application of native HNK‐1 glycan is limited by challenges in synthesis and rapid degradation in vivo [74]. To overcome these limitations, an HNK‐1 glycomimetic peptide was developed to mimic the biological functions of native carbohydrate, reproducing its ability to mediate cell−cell interactions that favor axon elongation. This peptide binds to motor neurons in vitro and preferentially promotes neurite outgrowth and extension from motor axons compared with sensory neurons [70]. When applied within a polyethylene cuff to reconstruct the continuity of a transected femoral nerve, the HNK‐1 glycomimetic peptide markedly enhanced functional recovery—quadricep muscle function reached 93% of normal within three months, whereas restoration in the control groups was less complete (71%−76%). In addition, treatment with the peptide led to larger motoneuron somata, improved axonal myelination in the quadriceps nerve, and greater precision of target reinnervation, while lesion‐induced motoneuron death was reduced by approximately 20%−25% [72]. Furthermore, the HNK‐1 glycomimetic peptide was covalently bonded to oligomeric type I collagen (Col I) via the crosslinker 1‐ethyl‐3‐(3‐dimethylaminopropyl) carbodiimide (EDC) to generate a hydrogel. The glycomimetic peptide maintained its bioactivity after functionalization on the Col backbone, and the grafted HNK‐1 glycomimetic peptide promoted motor neurite branching in vitro [74]. The HNK‐1 glycomimetic peptide‐coupled Col successfully bridged a 5 mm gap in a mouse femoral nerve injury model, enhancing motoneuron targeting and axonal myelination and ultimately facilitating functional recovery [75].

Cell−ECM interactions regulate axon elongation by providing mechanical guidance and biochemical cues essential for PNR. These interactions are mediated by ECM proteins, such as laminins, fibronectin, and Cols, which bind to integrins and other CAMs on the neuronal surface [76, 77]. Cell adhesion peptides derived from ECM proteins have been developed to simulate these interactions by mimicking key active sites of ECM proteins, often integrin‐binding motifs, and activating adhesion‐associated intracellular signaling pathways—most notably those related to focal adhesion assembly and cytoskeletal remodeling—that regulate growth cone motility [78]. Numerous cell adhesion peptides have been identified or synthetically developed. Among them, the most frequently utilized in tissue engineering studies are the fibronectin‐derived peptide RGD (89%) and laminin‐derived peptides IKVAV (6%) and YIGSR (4%) [79]. However, in the context of PNR, recent studies have more prominently employed IKVAV rather than YIGSR or RGD, and IKVAV has shown a powerful ability to promote axon elongation. When incorporated at 8.0 mol peptide per mole of fibrinogen, IKVAV, YIGSR, and RGD produced different effects on neurite extension within fibrin matrices: Extension was enhanced by both IKVAV and YIGSR, with IKVAV producing the more potent effect, but reduced by RGD. Therefore, the IKVAV peptide is discussed in detail in this section [80].

**FIGURE 2:**
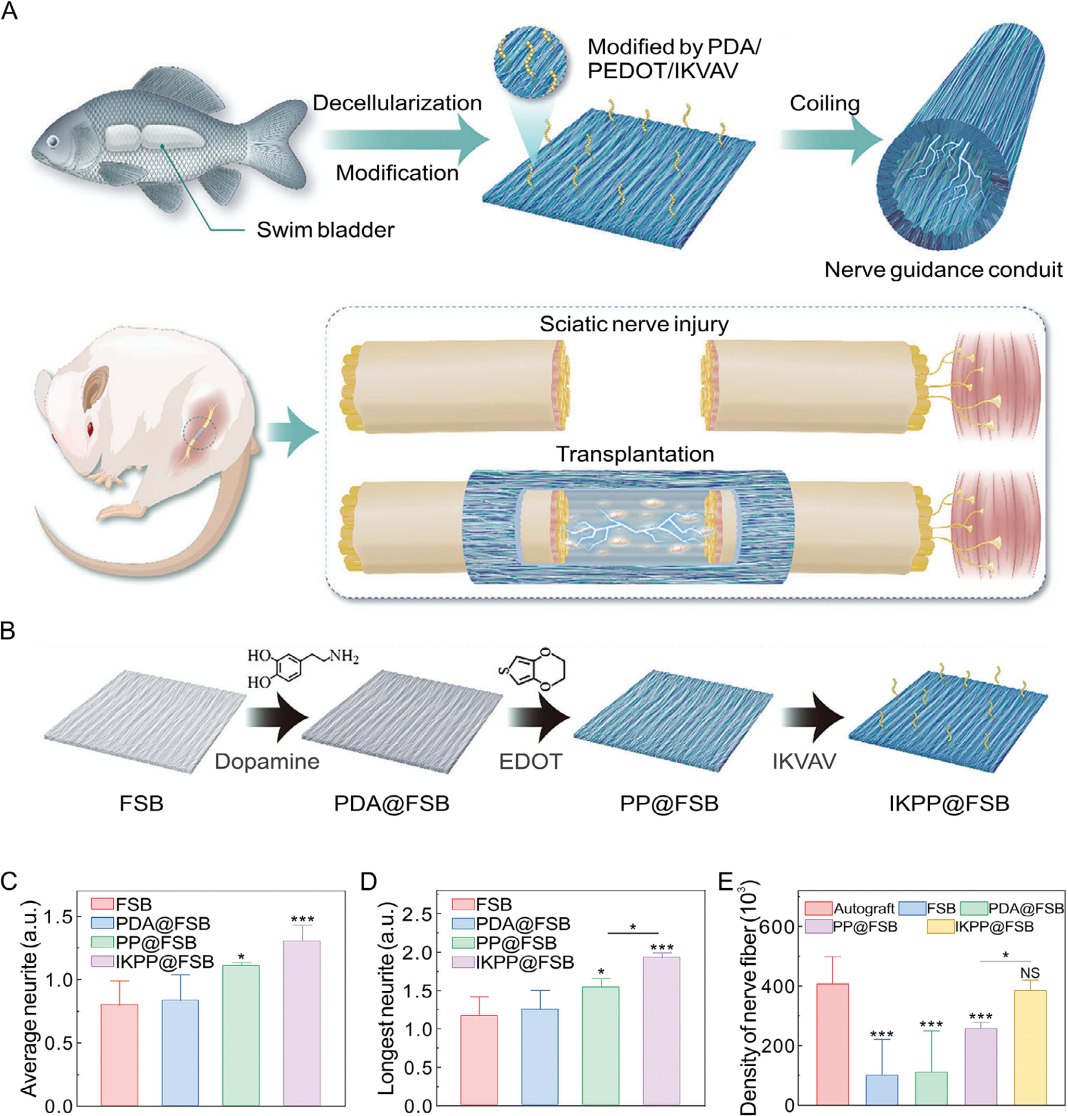
IKVAV peptide‐incorporated FSB as conductive nerve conduits. (A) IKVAV peptide‐incorporated FSB as a conductive nerve conduit for PNR. (B) Fabrication process of FSBs under different treatments. (C) Ratio of average neurite length to cell body diameter in each DRG cultured on FSB, PDA@FSB, PP@FSB, and IKPP@FSB. (D) Ratio of longest neurite length to cell body diameter of each DRG cultured on FSB, PDA@FSB, PP@FSB, and IKPP@FSB. (E) Statistical analysis of nerve fiber density per square micron in sciatic nerve regeneration. Reproduced with permission [86]. Copyright 2024, Wiley.

The IKVAV peptide is a pentapeptide motif originating from the laminin α‐1 chain [81]. Laminins are key ECM components in peripheral nerves. They are large heterotrimeric glycoproteins (400−900 kDa) composed of three subunits (α, β, and γ) that assemble into cross‐shaped structures [82]. Compared with full‐length laminin, short adhesive peptides derived from laminin, such as IKVAV, exhibit several advantages, including greater stability against denaturation and enzymatic degradation, higher incorporation density, lower risk of immune rejection, and feasibility for large‐scale chemical synthesis [83]. IKVAV represents one of the principal domains of laminin in regulating cellular behaviors. By interacting with the β1‐integrin subunit, IKVAV promotes neurite outgrowth, neuronal viability, and maturation [84, 85]. For example, Zhang et al. recently engineered a poly(3,4‐ethylenedioxythiophene) (PEDOT)‐coated, decellularized fish swim bladder (PP@FSB) modified with the IKVAV peptide as a conductive nerve conduit. The IKVAV peptide was immobilized to increase cell‐adhesion sites and improve the biocompatibility of FSB substrates by pre‐coating with polydopamine (Figure 2A,B). Compared with the unmodified PP@FSB, the IKVAV‐modified PP@FSB significantly enhanced the average and maximal neurite length‐to‐cell body diameter ratios in DRG neurons in vitro (Figure 2C,D) and exhibited greater nerve fiber density in vivo (Figure 2E) [86].

### 2.3 Suppression of Intrinsic Inhibitory Factors

Intrinsic inhibitory factors are crucial to the neuronal intrinsic properties that influence axonal elongation capacity [31, 87]. After PNI, the extracellular microenvironments accumulate growth‐inhibitory molecules, including chondroitin sulfate proteoglycans (CSPGs) in the ECM and MAG derived from myelin debris, hindering the regenerative capacity of peripheral axons [88]. RhoA, a small GTPase, has been proposed as a convergence point that transduces many extracellular inhibitory cues into intracellular signaling that restricts axon elongation [89, 90]. RhoA becomes activated in neurons after axonotmesis, where it triggers growth cone collapse and neuronal death at the lesion site through its downstream effector, ROCK. At the molecular level, activation of the RhoA/ROCK axis leads to actomyosin contractility and cytoskeletal reorganization within growth cones, thereby restricting axon elongation [87, 88]. Consequently, suppression of RhoA is widely recognized as a therapeutic strategy to promote axon elongation and PNR [87, 91]. Notably, RhoA can be inhibited by C3 exoenzyme from Clostridium botulinum (C3bot), a bacterial toxin, and the type III intermediate filament protein vimentin serves as a membrane binding partner that facilitates C3bot's access to damaged neurons [92, 93]. C3bot and its peptidic fragments have been shown to promote axonal and dendritic outgrowth both in vitro and in vivo [94]. Following the discovery that a 29‐amino acid fragment (C3^154−182^) derived from C3bot enhanced axonal and functional recovery after spinal cord injury, Huelsenbeck et al. investigated the effect of a 26‐amino acid fragment (C3^156−181^), the shortest active peptide derived from C3bot, on PNR. A single topical application of the C3^156−181^ peptide promoted axonal elongation toward motor targets, enhanced axonal maturation, and accelerated motor recovery, likely through RhoA inactivation. Notably, treatment with C3^156−181^ produced greater improvement in PNR than the wild‐type C3bot protein, indicating a more neuron‐specific mode of action [95].

G3BP1 granules are newly identified intrinsic inhibitory factors of axon elongation. G3BP1 functions as a core component and essential regulatory factor of stress granules, which are emerging therapeutic targets and are membraneless organelles formed in the cytoplasm via liquid−liquid phase separation [96, 97, 98]. Previous studies indicate that axonal G3BP1 granules hinder PNS axon regeneration by sequestering mRNAs and inhibiting their translation within injured axons. A cell‐permeable G3BP1 B‐domain peptide (190−208) was synthesized and shown to disassemble G3BP1 granules, thereby enhancing axon growth in cultured sensory and cortical neurons [99]. To assess its in vivo efficacy, the peptide was injected just proximal to the injury site within the perineurium two days after sciatic nerve crush. Treated animals exhibited a marked increase in axon regeneration seven days post‐injury compared with controls. Mechanistically, the functional motif of the G3BP1 peptide (190−208) was found to consist of alternating acidic residues (Glu or Asp) and Pro repeats, which disrupts G3BP1‐mediated stress granule assembly, thereby releasing sequestered axonal mRNAs and restoring local protein synthesis required for axon elongation [99, 100].

### 2.4 Mediation of Synergistic Effects

In addition to their direct bioactivity in promoting axon elongation, specific peptides actively target neurons, thereby enhancing the delivery efficiency of coupled therapeutic cargos and producing synergistic regenerative effects. A wide range of drugs and other cargos loaded within biomaterials must reach and penetrate neurons to exert their effects, a challenging process because neurons are non‐phagocytic compared with glial cells [101]. Compared with approaches that rely on nanoparticles that inherently target neurons or on disease‐related pathological features, peptide‐based active targeting is generally more specific and versatile, thus accommodating diverse therapeutic requirements and improving drug‐delivery efficiency [102]. In this strategy, peptides serve as ligands that recognize neuron‐specific surface targets, directing the intracellular delivery of therapeutics and supporting PNR [101].

**FIGURE 3:**
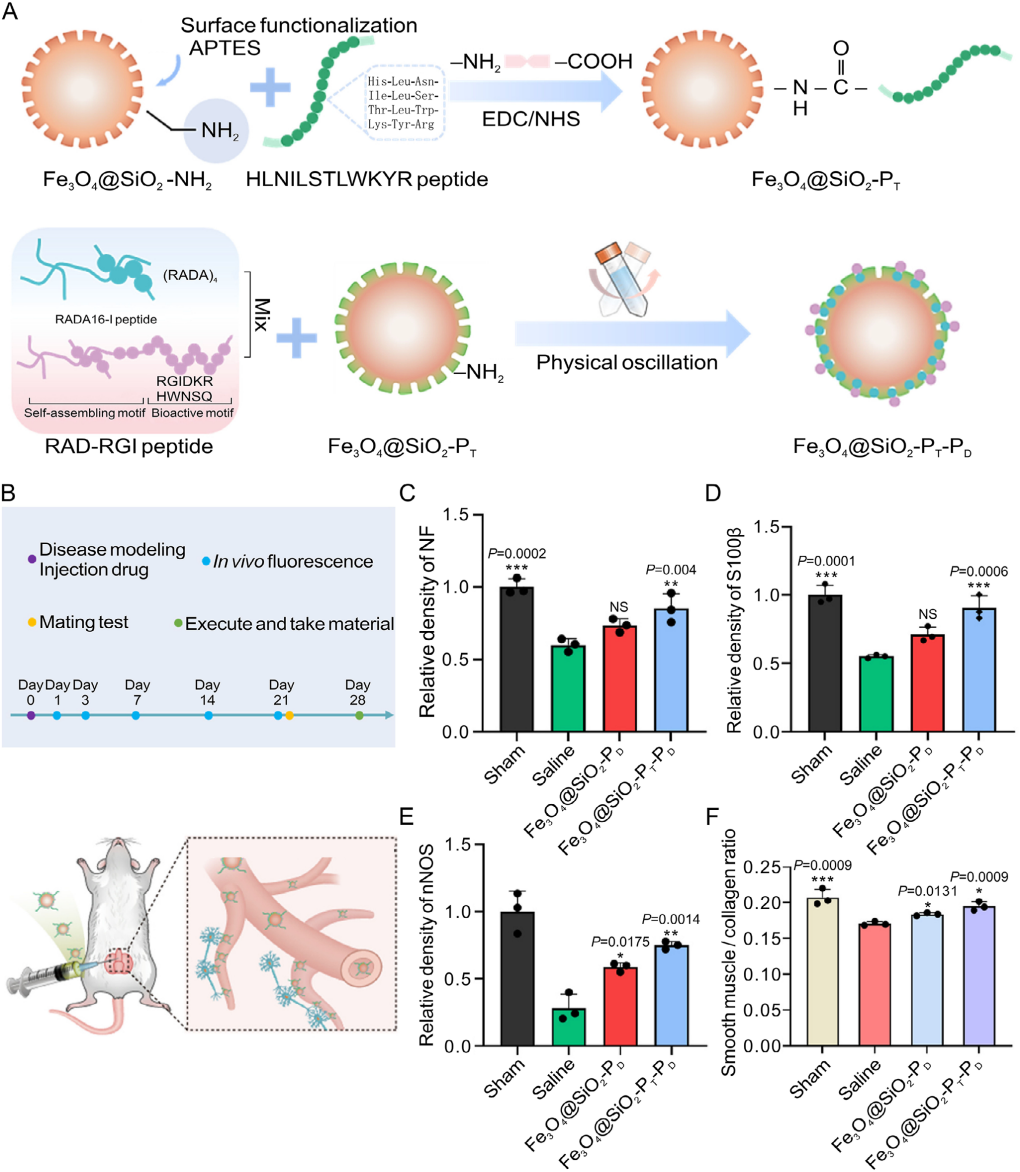
Magnetic mesoporous silica nanoparticles functionalized with Tet‐1 and loaded with RGI peptides for targeted repair of cavernous nerve injury. (A) Preparation of Fe_3_O_4_@SiO_2_‐P_T_‐P_D_. (B) Schematic overview of experimental procedure. Comparison of NF (C), S100β (D), and nNOS (E) expression in corpus cavernosum between Sham and experimental groups. (F) Mean densitometry ratio between smooth muscle and Col contents. Reproduced with permission [105]. Copyright 2024, Elsevier.

The Tet‐1 peptide is often investigated for its potential in actively target neurons. This peptide consists of 12 amino acids and is derived from a neurotoxin (tetanus toxin) through phage display [103]. It binds to molecules, such as GT1b gangliosides and sphingophospholipids, which are highly expressed on neurons. Through these interactions, Tet‐1 enables neuron‐specific membrane engagement and intracellular trafficking, thereby allowing the preferential delivery of therapeutic cargos to neuronal cell bodies. Effective uptake of Tet‐1 peptide and retrograde axonal transport in vitro and in vivo have been shown by Federici et al. [104]. Tet‐1 has been employed in many studies to modify the surface of nanoparticles to improve targeting efficiency, and it has demonstrated the ability to mediate axonal uptake and delivery to neuronal cell bodies (soma). For example, Liang et al. covalently grafted the Tet‐1 peptide onto magnetic mesoporous silica nanoparticles loaded with RADA16‐I/RAD‐RGI mixed peptide drugs for targeted neuroregenerative therapy (Figure 3A). In fluorescence assays, PC‐12 cells treated with Tet‐1‐grafted nanoparticles exhibited 3.5‐fold higher fluorescence intensity compared with those treated with ungrafted nanoparticles. These Tet‐1 peptide‐incorporated, neuro‐targeted nanoparticles were injected into the injury site of a rat model with bilateral cavernous nerve defects (Figure 3B). Tet‐1 grafting markedly improved targeting of the injured nerve and enhanced drug accumulation at the lesion site. This was accompanied by significantly increased expression of NF, S100β, and nNOS, indicating enhanced nerve regeneration (Figure 3C–E). Moreover, the elevated mean densitometry ratio of smooth muscle to Col content suggested improved smooth muscle repair and reduced fibrosis, ultimately contributing to improved functional nerve recovery (Figure 3F) [105].

The RVG29 peptide, originating from rabies virus glycoprotein, binds specifically to neurons that express the nicotinic acetylcholine receptor (nAChR). Consistent with the neurotropic properties of rabies virus glycoprotein, RVG29 enables receptor‐mediated neuronal targeting and internalization, allowing loaded cargos to access neurons preferentially [106, 107]. It was combined with poly(ethylene glycol)‐*block*‐poly(lactic acid) (PEG‐*b*‐PLA) to generate ligands on nanoparticle surfaces, referred to as polymersomes, which were loaded with the near‐infrared dye AlexaFluor647. The RVG29 peptide tends to bind neural‐specific receptors selectively, and its incorporation resulted in elevated AlexaFluor647 fluorescence at the injury site following intranerve or intramuscular injection compared with the untagged control. RVG29‐tagged nanoparticles exhibited enhanced specificity for targeting neurons and enabled the delivery of cargo into neurons [108]. These findings suggest that replacing AlexaFluor647 with an axon growth‐promoting drug may further enhance axonal extension via this synergistic delivery system.

Three peptide ligands, designated DRG homing peptides, were identified through biopanning of a phage display library for selective targeting of DRG neurons, the initiating neurons of sensory pathway [109]. These DRG‐homing peptides mediate targeted delivery of therapeutic genes to DRG neurons. Mechanistically, these peptides function as targeting ligands that redirect vector tropism by mediating preferential binding and internalization into DRG neurons, thereby enabling efficient and selective cargo delivery [110]. Terashima et al. genetically modified an adenovirus (Ad) to generate a helper virus (HV) containing DRG homing peptides, referred to as DRG‐targeted helper‐dependent Ad (HDAd). DRG homing peptides were introduced into the BspEI region of capsid fiber protein in the modified Ad. The resulting HDAd efficiently and specifically transduced DRG neurons in vitro and in vivo. Of the three DRG homing peptides assessed, HV‐DRG1 showed the highest transduction efficiency, followed by HV‐DRG2 and HV‐DRG3. A single injection of DRG‐targeting HDAd expressing β‐hexosaminidase restored enzyme activity and function of sensory neurons in a mouse model of Sandhoff disease [110], suggesting that these DRG homing peptides possess the potential for synergistically treating sensory nerve damage or selectively delivering cargo to sensory neurons.

## 3 Peptides Enhance Schwann Cell Support

During PNR, SCs act as central coordinators of structural and trophic support, as summarized in Scheme 2. As the principal glial cells of PNS, SCs normally generate the myelin sheath that insulates axons and accelerates impulse transmission, while also providing metabolic support critical for maintaining axonal integrity [111, 112, 113]. After PNI, SCs support PNR through multiple mechanisms, including reprogramming into a stem‐like repair phenotype, migrating to form guidance pathways for axonal regrowth, and subsequent remyelinating that enables axonal maturation [114]. In the context of peptide‐based strategies for targeting SC support, however, existing studies have predominantly focused on two mechanisms—SC reprogramming and SC migration—while remyelination is more often considered a downstream outcome rather than a primary intervention target [45, 115]. This tendency may partly reflect the fact that remyelination occurs at a relatively late stage of regeneration and may require intervention during a delayed phase of repair, which is inherently challenging to achieve with localized biomaterial‐based strategies. Moreover, the early regenerative phase depends on SC dedifferentiation into a repair phenotype, and premature promotion of myelination may potentially disrupt this coordinated transition. Accordingly, this section centers on peptide‐induced SC reprogramming and migration as the main modes by which peptide incorporation enhances SC support for nerve regeneration.

### 3.1 Induction of Schwann Cell Reprogramming

After PNI, SCs exhibit remarkable plasticity and reprogram into a repair phenotype driven mainly by activation of the transcription factor c‐Jun [116, 117]. These repair SCs downregulate myelin‐associated genes and upregulate repair‐supportive programs in regions distal to the injury. They release trophic factors, such as GDNF, BDNF, NT‐3, NGF, and vascular endothelial growth factor (VEGF), which promote neuronal survival and axonal elongation, and they secrete cytokines, including tumor necrosis factor‐α (TNF‐α), interleukin‐1α (IL‐1α), IL‐1β, and monocyte chemoattractant protein‐1 (MCP‐1) that participate in the innate immune response [28]. However, this repair program progressively fails in aging animals and during chronic denervation, due to slow axonal regrowth [118]. Thus, the acquisition and sustained maintenance of this reprogrammed repair phenotype are prerequisites for efficient nerve regeneration, particularly in aging or chronically denervated microenvironments where SCs tend to adopt a senescent state [119, 120]. Accordingly, strategies that promote or stabilize SC reprogramming represent critical therapeutic approaches for improving PNR.

Low‐density lipoprotein receptor‐related protein‐1 (LRP1) signaling has emerged as a critical target for inducing SC reprogramming. LRP1, an endocytic and cell‐signaling receptor, is markedly upregulated in SCs following PNI, promoting their survival and repair programs [121]. Ligand binding to LRP1 activates cell signaling, including MAPK‐ERK, the transcription factor c‐Jun, and the PI3K‐Akt pathway, and LRP1 signaling has been shown to inhibit the unfolded protein response. Kim et al. developed a 22‐amino acid peptide, referred to as peptide 2, an LRP1 agonist that activates SC reprogramming. This peptide is derived from the hemopexin domain (PEX) of matrix metalloproteinase 9 (MMP‐9), a known LRP1 ligand in the injured peripheral nerve. The PEX of MMP‐9 facilitates LRP1 activation rather than relying on MMP‐9 protease activity. Therefore, peptide 2 offers novel therapeutic potential by mimicking PEX's role in activating LRP1 without exhibiting proteolytic activity. It was demonstrated that peptide 2 binds to the LRP1 binding domains CCR II and CCR IV, activating Akt and ERK1/2 signaling in primary human SCs. Intraneural injection of peptide 2 into crush‐injured sciatic nerves in mice increased c‐Jun activation by more than 2.5‐fold, indicating that peptide 2 induces LRP1‐mediated SC reprogramming in vivo [122]. Although this study primarily elucidates the molecular mechanism of peptide 2‐mediated LRP1 activation and provides limited in vivo functional or behavioral characterization, peptide 2 nevertheless holds translational potential for enhancing SC reprogramming‐based nerve repair, particularly in challenging contexts, such as aging or chronic denervation.

### 3.2 Promotion of Schwann Cell Migration

After PNI, SCs migrate along the preserved basal lamina tubes of distal nerve stump and, after transection, into the fibrin “bridge” spanning the injury gap, where they form structures known as the bands of Büngner, which serve as guidance pathways for regenerating axons. In addition, SCs secrete signaling molecules that recruit macrophages, support neuronal survival, activate mesenchymal stem cells, and coordinate interactions with other cell types [123]. In vivo axonal regeneration is constrained by the extent to which SCs migrate into the gap between the proximal and distal stumps [124]. Several peptides derived from CAMs and ECM proteins, as mentioned above, have been shown to promote SC migration, and the gastric inhibitory peptide/gastric inhibitory peptide receptor (GIP/GIPR) signaling axis also contributes to SC migratory activity.

The KHI peptide serves as a homophilic binding domain of neural CAM (N‐CAM), a key member of the Ig‐CAM family, and is widely studied for its interactions with other transmembrane proteins, such as integrins [69, 125]. Interestingly, the KHI peptide is capable of selectively modulating gliocyte and fibroblast adhesion to material surfaces by increasing the adhesion of gliocytes instead of fibroblasts [126]. Leveraging this attribute, Ren et al. designed a surface incorporating a complementary gradient of KHI peptides and PDMAPS (a zwitterionic polymer with antifouling properties). SCs exhibited preferential orientation and approximately two‐fold improved directional migration on the gradient surface toward regions of increased KHI peptide density and lower PDMAPS density, whereas fibroblasts displayed random migration and a 40% decrease in migration rate compared with their behaviors on glass. Notably, these findings were obtained at the in vitro level, and in vivo evidence remains to be established. Therefore, this peptide shows potential for facilitating nerve regeneration while concurrently inhibiting fibrosis [127]. However, it should be noted that, although these effects are promising, the molecular mechanisms underlying the selective cellular responses to KHI remain poorly understood and warrant further investigation.

YIGSR, an ECM‐derived cell adhesion peptide, exerts notable effects on SC migration. In one study, YIGSR (derived from the laminin β1 chain) and RGD (derived from the fibronectin domain FNIII) peptides were immobilized onto substrates to form a concentration gradients using a controlled vapor deposition technique, followed by covalent peptide binding via a thiol‐ene reaction. While tethered RGD peptides enhanced SC adhesion and proliferation, RGD gradients had little effect on SC directional migration. In contrast, although YIGSR supported lower SC adhesion than RGD, YIGSR gradients effectively guided SC migration along the concentration gradient [124]. Despite the widespread adoption of RGD in tissue engineering applications, it does not appear to be the optimal peptide for promoting SC migration. In another study, the CYIGSR peptide was bio‐orthogonally linked to the methacrylate groups of chitosan conduits via a thiol‐ene reaction, generating an esterase‐responsive release system. In a rat sciatic nerve defect model, these CYIGSR‐functionalized conduits resulted in markedly improved sciatic nerve functional recovery and greater target muscle weight compared with non‐functionalized conduits [128]. Notably, although these studies demonstrate clear functional benefits, the molecular mechanisms by which YIGSR gradients preferentially guide SC migration remain to be systematically elucidated.

**FIGURE 4:**
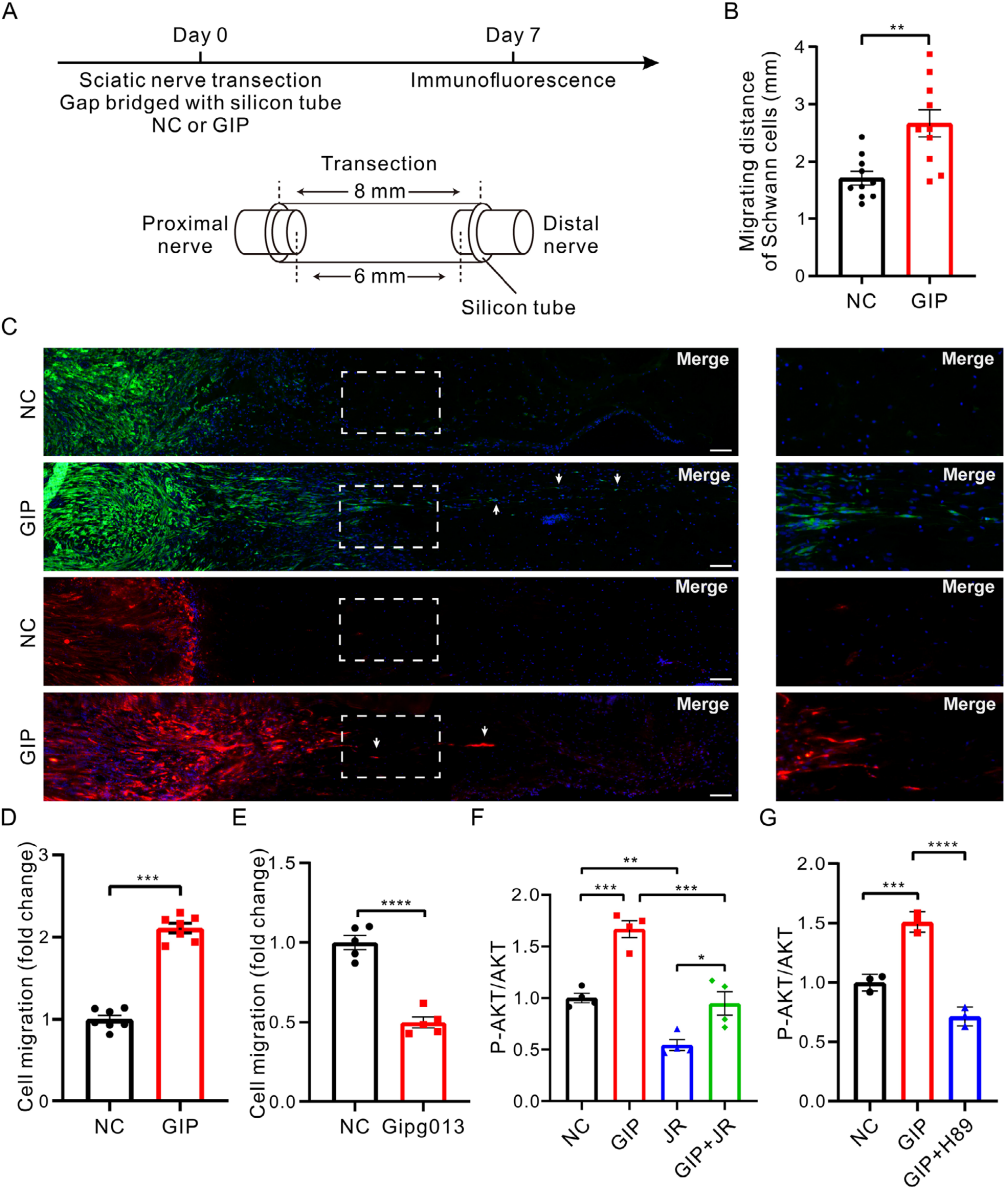
Activation of the GIP/GIPR axis via sonic hedgehog signaling promotes the bridging of gapped nerves in sciatic nerve injury. (A) Schematic diagram of sciatic nerve injury model. GIP promoted SC migration and axonal extension across the gap. (B) Average migration distance of SCs. (C) Representative immunostaining images of sciatic nerve longitudinal sections from rats injected with a negative control or GIP. Rat sciatic nerve segments are collected seven days after nerve transection injury. Boxed areas exhibit a higher magnification. White arrows mark SCs that have migrated and regenerated axons. Red color indicates SCG10, green color indicates S100β, and blue color indicates the nucleus. Scale bar  =  100 µm. (D) GIP significantly enhanced SC migration. Representative images and quantification data of Transwell migration assay. (E) GIPR blocking with Gipg013 significantly inhibited SC migration. Representative images and quantification data of Transwell migration assay. (F) GIP‐induced phosphorylation of AKT at Ser 473 via mTORC2. SCs were stimulated with GIP in the presence or absence of JR‐AB2‐ 011 or remained untreated as indicated, and phosphorylation of AKT Ser473 was analyzed. Total AKT levels are shown as a loading control. (G) Activation of mTORC2 mediated by GIP is PKA‐dependent. PKA inhibitor H89 inhibited the upregulation of AKT Ser473 phosphorylation upon GIP stimulation. Reproduced with permission [132]. Copyright 2023, Wiley.

The GIP/GIPR axis, in addition to its primary role in regulating postprandial blood glucose levels and energy storage [129], facilitates PNR by promoting SC migration and axonal elongation. The GIPR is a GPCR belonging to the glucagon receptor family, and it increases cAMP levels upon GIP activation [130]. Under normal conditions, GIP and GIPR levels in SCs are low. However, they increase significantly after injury, indicating their essential role during PNR [131]. In a rat model of sciatic nerve defect, a silicone conduit filled with recombinant GIP peptide was used to bridge the nerve gap (Figure 4A). The application of GIP‐loaded silicone tubes markedly enhanced SC migration and facilitated SC cord formation during regeneration, thereby promoting nerve fiber growth (Figure 4B,C), as shown by immunostaining for the SC marker S100β and axonal marker SCG10. To further investigate the underlying mechanism, an in vitro Transwell migration assay was performed. The results showed that GIP significantly promoted SC migration (Figure 4D), whereas antagonism of GIPR using the Gipg013 antibody reduced the number of migrating SCs (Figure 4E). Increased phosphorylation of AKT at Ser‐473 following GIP stimulation, and its reduction by the mTORC2 inhibitor JR‐AB2‐011 (JR), indicated that mTORC2 activity is essential for GIP‐induced SC migration (Figure 4F). Furthermore, GIP‐induced phosphorylation of AKT (Ser‐473) was attenuated by the PKA inhibitor H89, suggesting that GIP promotes SC migration via the PKA‐mTORC2 signaling pathway (Figure 4G) [132].

## 4 Peptides Modulate Inflammatory Microenvironments

Inflammatory microenvironments represent one of the earliest and most dynamically regulated components of PNR, as outlined in Scheme 2. Following PNI, the inflammatory microenvironments evolve through distinct yet interconnected phases [32, 133]. In the early stage, a pro‐inflammatory response facilitates axonal and myelin debris clearance, thus helping to create a permissive microenvironment for regeneration. Subsequently, the inflammatory milieu gradually shifts to an anti‐inflammatory (pro‐regeneration) state, during which immune components with anti‐inflammatory activity promote tissue repair [134]. This stage is characterized by M2 macrophage polarization and the release of anti‐inflammatory cytokines. However, this pro‐regenerative process is often hindered by excessive inflammation, leading to cell necrosis, apoptosis, and neuropathic pain [135]. Therefore, researchers seek strategies to transform excessively inflammatory microenvironments into anti‐inflammatory states to enhance PNR [136].

**FIGURE 5:**
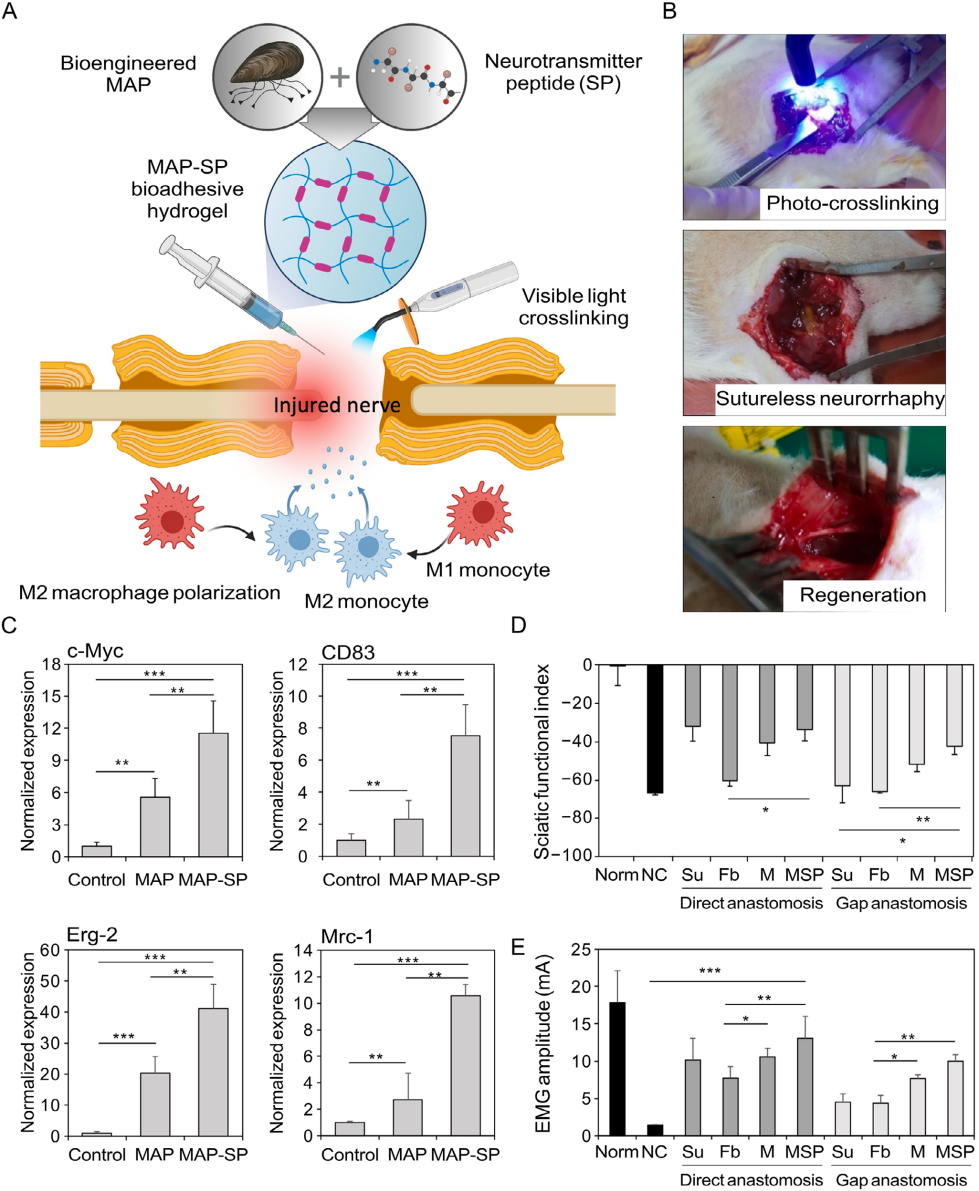
Sutureless neurorrhaphy system using a macrophage‐polarizing in situ visible light‐crosslinkable adhesive protein hydrogel for functional nerve regeneration. (A) Macrophage‐polarizing in situ visible light‐crosslinkable adhesive protein hydrogel for functional sutureless neurorrhaphy. (B) Sutureless in situ visible light‐crosslinkable MAP‐SP bioadhesive hydrogel neurorrhaphy procedure for nerve tissue regeneration. Hydrogel gelation was performed by illumination with a blue LED dental curing lamp from a distance of 50 mm for 60 s. (C) M2 macrophage‐related genotypic markers of the c‐Myc, CD83, Erg‐2, and Mrc‐1 genes were quantified using qRT‐PCR. Assessment of functional recovery of sciatic nerve using (D) SFI and (E) EMG. Reproduced with permission [140]. Copyright 2022, Elsevier.

Substance P (SP) is an 11‐amino acid neuropeptide that mediates interactions between neurons and immune cells. It exerts biological and immunological effects through neurokinin receptors (NKRs), particularly the high‐affinity neurokinin 1 receptor (NK1R), and regulates immune cell proliferation, mobilization, and cytokine production [137, 138]. In the context of PNR, SP acts as an inducer of M2 macrophage polarization and plays an anti‐inflammatory role. Mechanistically, SP signals through NK1R on macrophages, leading to activation of PI3K‐Akt‐mTOR pathways that drive a tissue‐repairing (M2‐like) polarization program, thereby contributing to an inflammatory milieu conducive to regeneration [139, 140]. Cheong et al. genetically fused SP to a mussel adhesive protein (MAP)‐based bioadhesive hydrogel to create a sutureless neurorrhaphy system via in situ visible light crosslinking (Figure 5A,B). M2 macrophage polarization on the MAP‐SP‐coated surface was markedly enhanced, as evidenced by the upregulated expression of phenotypic markers including c‐Myc, CD83, Erg‐2, and Mrc‐1 (Figure 5C). In rat sciatic nerve defect and transection models, the SP‐incorporated bioadhesive hydrogel enabled effective sutureless anastomosis and promoted anti‐inflammatory M2 macrophage polarization that supported tissue remodeling, while increased sciatic functional index (SFI) and electromyography (EMG) amplitude demonstrated significant improvement in functional nerve regeneration (Figure 5D,E) [140].

The stromal cell‐derived factor 1 (SDF‐1), also denoted CXCL12, is an important chemokine that orchestrates the migration and activation of multiple cell types, including leukocytes, hematopoietic progenitor and stem cells, and ECs, primarily by interacting with its receptors, CXCR4 and ACKR3. This signaling axis plays crucial roles in inflammation, hematopoiesis, and angiogenesis [141, 142]. Numerous studies have examined the incorporation of SDF‐1 into hydrogels or scaffolds to achieve localized delivery and promote injury repair [143]. However, the native SDF‐1 protein has a limited half‐life and is susceptible to degradation. Consequently, a mimetic peptide of SDF‐1α has been developed that preserves the receptor‐activating domain of the native protein [144, 145]. Mechanistically, the SDF‐1α mimetic peptide recapitulates CXCL12‐CXCR4‐mediated chemokine signaling, which modulates macrophage polarization and promotes angiogenesis, thereby supporting a pro‐regenerative microenvironment [141, 142, 143]. Recently, a porous methacrylated gelatin hydrogel was fabricated to encapsulate and continuously release the SDF‐1α mimetic peptide within a zeolitic imidazolate framework‐8 nanocarrier. This peptide‐loaded hydrogel effectively promoted M2 macrophage polarization, attenuated inflammation, and enhanced neovascularization and remyelination, ultimately facilitating nerve regeneration in rat sciatic and facial nerve crush injury models. Notably, the ability of SDF‐1α mimetic peptide nanoparticle to modulate M2 macrophage polarization was dose‐dependent, with 10 µg mL^−1^ producing the most substantial reductions in CD86 and iNOS markers and the most pronounced increase in CD206 [146].

Erythropoietin (EPO), traditionally known for facilitating erythropoiesis in the hematopoietic system via binding to the (EPOR)_2_ homodimer, has also been identified as an anti‐inflammatory cytokine functioning via binding to the tissue protective receptor (TPR) complex in non‐hematopoietic tissues [147, 148]. However, prolonged use of EPO may have adverse effects, including thrombosis and hypertension. To overcome this limitation, non‐hematopoietic EPO analogs that selectively activate TPR without engaging (EPOR)_2_ have been developed. Among them, the ARA290 peptide has demonstrated notable neuroprotective effects. Mechanistically, ARA290 activates the TPR, a heteroreceptor comprising EPOR and the β‐common receptor, leading to Janus kinase 2 (JAK2)‐dependent cytoprotective signaling and attenuation of pro‐inflammatory responses, thereby reducing neuroinflammation without inducing erythropoiesis [149, 150]. For example, ARA290 administered for several weeks after spared nerve injury provided effective and sustained relief of PNI‐induced neuropathic pain for up to 15 weeks. This effect depended on the β‐common receptor, a component of the TPR complex, and was attributed to the anti‐inflammatory properties of ARA290 [150].

The SOCS1 protein is a member of the suppressors of cytokine signaling family, which serves as an intracellular negative regulator of pro‐inflammatory cytokine signaling [151]. SOCS1 inhibits the JAK‐STAT pathway by directly binding to activated JAKs on cytokine receptors via the SH2 domain, thus downregulating interferon‐γ (IFN‐γ), TNF‐α, and IL‐1β, as well as inhibiting its own production [152]. In injured mouse sciatic nerves, macrophages were identified as the primary source of SOCS1 expression, which was inversely correlated with the phosphorylation of JAK2 and STAT3 signaling proteins and with production of the pro‐inflammatory cytokines TNF‐α and IL‐1β. The tyrosine kinase inhibitor peptide (Tkip) is a SOCS1 mimetic peptide that binds to the autophosphorylation site of JAK2 [153]. In a sciatic nerve cut/ligation injury model (in which the nerve was transected and the cut ends ligated to prevent axon regeneration)—a model characterized by reduced SOCS1 levels and heightened inflammation with increased macrophage infiltration and prolonged IL‐1β and TNF‐α expression—treatment with Tkip for two weeks suppressed pro‐inflammatory cytokine signaling, including IL‐1β, and reduced macrophage numbers in the distal nerve segment by approximately 30% [154].

Although immune‐modulating strategies predominantly focus on promoting anti‐inflammatory responses, the potential benefits of accelerating the early pro‐inflammatory phase have received increasing attention [155, 156, 157, 158]. The early pro‐inflammatory process facilitates the degradation and clearance of injured and degenerative distal nerve fibers that otherwise impede regeneration, a hallmark of Wallerian degeneration [135]. Immune cells, such as macrophages and neutrophils, together with associated chemokines and cytokines including LIF, TNF‐α, and MCP‐1, orchestrate the local pro‐inflammatory microenvironments [26, 159]. Accelerating this initial inflammatory phase promotes debris removal and allows an earlier transition to the subsequent anti‐inflammatory phase, thus improving regenerative outcomes [155].

**FIGURE 6:**
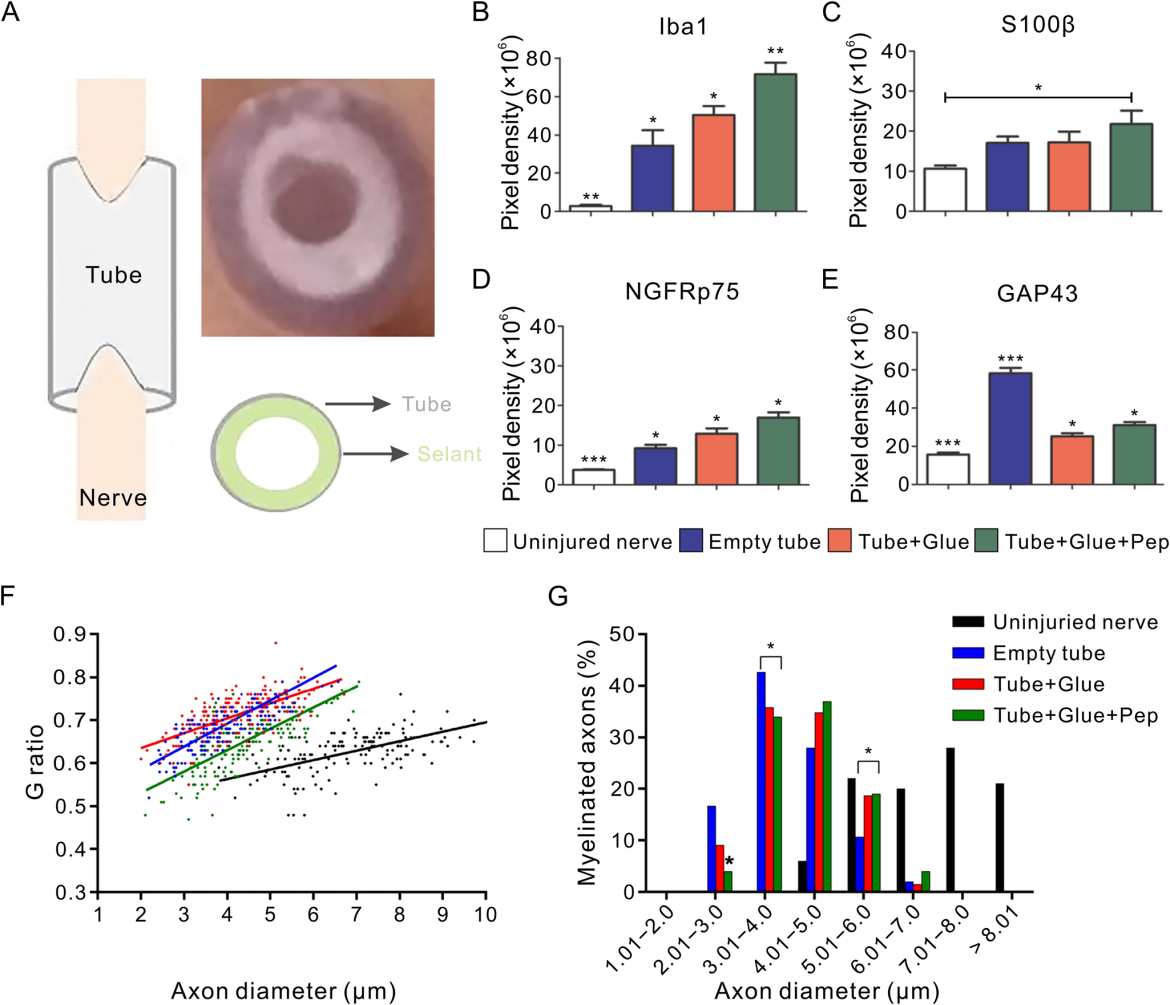
TNF‐mimetic peptide mixed with fibrin glue improves PNR. (A) Tubular prostheses are placed on the nerve following neurotmesis. BthTL fibrin sealant is distributed around the tube's internal walls. Quantification of Iba1 (B), S100β (C), NGFRp75 (D), and GAP43 (E) immunostaining density from uninjured and regenerated nerve segments of Empty Tube, Tube+Glue, and Tube+Glue+Pep groups. (F) G ratio against axon diameter (µm). (G) Distribution of myelinated fibers is similar for all axotomized groups, with a significant decrease in small fibers in Tube+Glue+Pep nerves compared with Empty Tube and Tube+Glue, respectively. Reproduced with permission [162]. Copyright 2021, Elsevier.

TNF‐α is an important pro‐inflammatory cytokine that triggers the activation cascade of other cytokines and growth factors, in part through TNF receptor‐mediated activation of nuclear factor κ‐B (NF‐κB) and MAPK pathways [160]. Soon after PNI, SCs, resident macrophages, and mast cells increase TNF‐α synthesis and release, leading to immense macrophage recruitment and continued Wallerian degeneration [161]. To enhance TNF bioavailability and leverage its role during the early inflammatory response, the Seq ID n° 2 TNF‐mimetic peptide (patent application BR 10 2020 007,233) was examined in combination with the fibrin glue BThTL (patent registration PI‐0406273−6) as a filler inside a silicon tube bridging an acute sciatic nerve defect in rats (Figure 6A). Peptide incorporation resulted in pronounced macrophage recruitment at the injury site, as indicated by elevated Iba1 expression (Figure 6B). In addition, it increased anti‐S100β, anti‐NGFRp75, and anti‐GAP43 immunostaining, reflecting enhanced SC activity, a pro‐regenerative phenotype, and increased growth cone numbers (Figure 6C–E). The g‐ratio against axon diameter analysis revealed that the peptide‐incorporation group most closely resembled the uninjured group (Figure 6F). Furthermore, the peptide‐incorporation group exhibited a greater proportion of large‐diameter myelinated axons (4−6 µm) and fewer small‐diameter myelinated axons (2−3 µm), demonstrating that Seq ID n° 2 peptide incorporation promoted enhanced axonal regeneration and remyelination (Figure 6G) [162].

Neutrophil peptide‐1 (NP‐1, also known as neutrophil defensin‐1) is an immunoregulatory peptide primarily released by neutrophils [163]. Its role in immune modulation is multifaceted: NP‐1 boosts bacterial phagocytosis by stimulating macrophages to upregulate TNF and IFN‐γ, but it also functions as a regulatory “molecular brake” that limits excessive macrophage‐driven inflammation, in part by suppressing macrophage mRNA translation and subsequent pro‐inflammatory cytokine production [164, 165]. These seemingly divergent effects are potentially related to disease type and stage, as well as dosage [166]. In the context of PNI, a single intraoperative administration of NP‐1 in a rat sciatic nerve crush model improved nerve conduction velocity and SFI at four weeks post‐surgery [163]. Subsequently, mechanistic studies demonstrated that NP‐1 promotes macrophage phagocytosis, proliferation, and migration, thus accelerating early pro‐inflammatory axonal debris clearance during Wallerian degeneration, as indicated by longer neurodegeneration distances at three and five days post‐injury using the CUBIC (Clear, Unobstructed Brain/Body Imaging Cocktails and Computational analysis) optical‐clearing technique, and facilitating the subsequent transition toward M2 macrophage polarization, ultimately enhancing PNR [155].

## 5 Peptides Induce Vascularization

Vascularization constitutes another indispensable component of the regenerative cascade illustrated in Scheme 2. The PNS requires a vascular network to deliver oxygen (O_2_) and nutrients to meet its substantial metabolic demands [167]. The blood supply of peripheral nerves can be categorized into two longitudinal systems: the extraneural vascular system, located in the perifascicular region, and the intraneural vascular system, located in the intrafascicular region [19]. Severe nerve injury frequently disrupts the local vasculature, and in the absence of vascular support, cell‐mediated repair processes are impaired because of insufficient O_2_ and nutrient availability. Furthermore, it has been proposed that newly established vasculature between the proximal and distal nerve stumps serves as a route guiding the migration of SCs alongside regenerating axons [27]. Collectively, vascularization is crucial for PNR.

Vascularization following PNI is primarily achieved via angiogenesis, a process in which new blood vessels sprout from pre‐existing ones and proliferate into areas lacking vascular supply. This process encompasses several events, including vasodilation, basement membrane degradation, EC migration, perivascular cell recruitment, EC proliferation, and blood vessel formation [167, 168].

**FIGURE 7:**
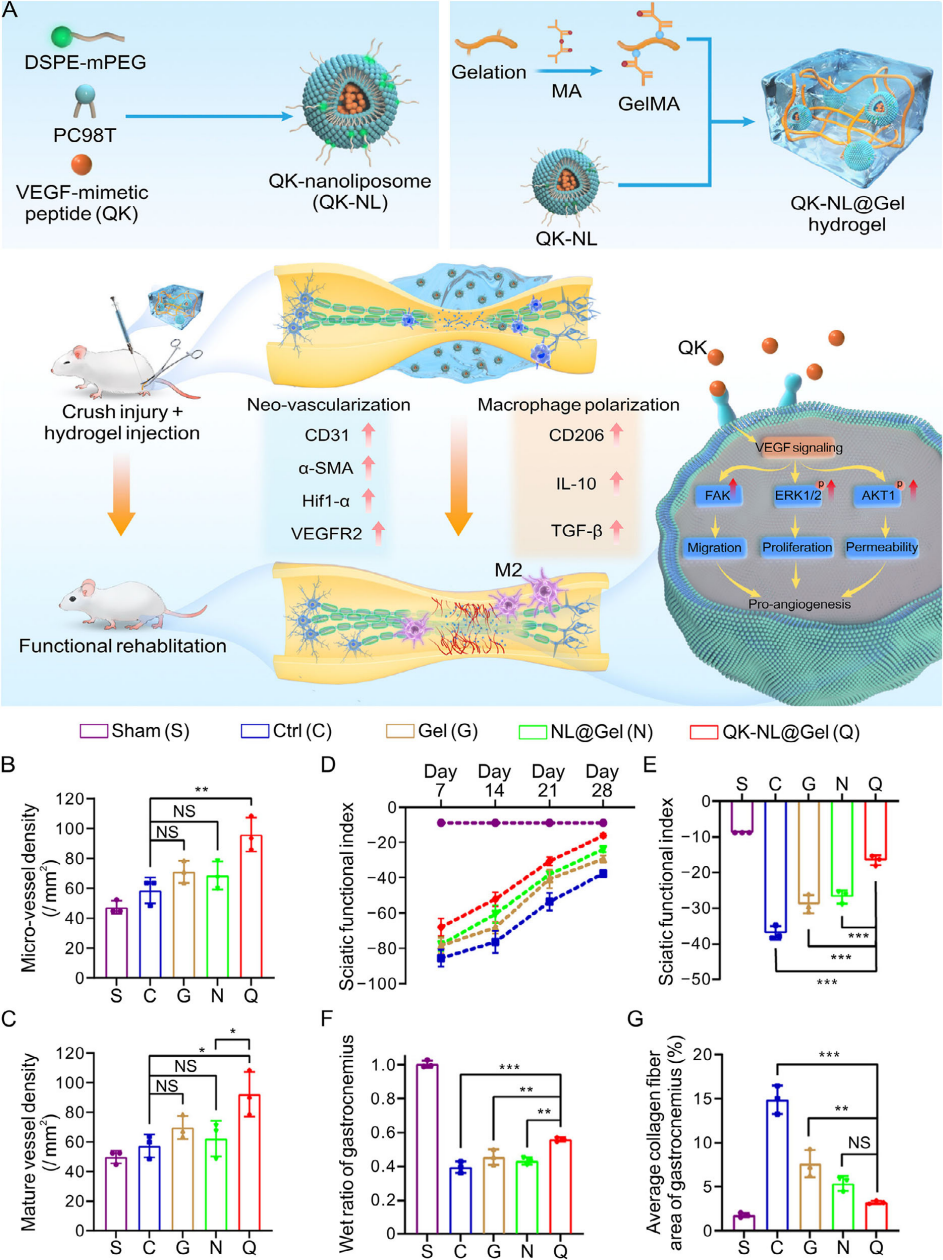
Injectable hydrogel encapsulated with VEGF‐mimetic peptide‐loaded nanoliposome promotes PNR. (A) Injectable VEGF‐mimetic peptide‐encapsulated nanoliposome (QK‐NL@Gel) hydrogel for PNI repair. Synthesis route of QK‐NL@Gel, promoting effect, and its regulatory mechanism on nerve regeneration. (B) Quantitative statistics of micro‐vessel density at 28 days post‐surgery. (C) Quantitative statistics of mature vessel density at 28 days post‐surgery. (D) Change in sciatic function index (SFI) at 7, 14, 21, and 28 days post‐surgery. (E) Statistical analysis of SFI at week 4 post‐surgery. (F) Statistical results of wet ratio of gastrocnemius muscle. (G) Statistical results of average Col fiber area of gastrocnemius muscle. Reproduced with permission [115]. Copyright 2023, Elsevier.

VEGF is the most commonly used promoter of angiogenesis because of its specific mitogenic effects on ECs [115, 169]. VEGF promotes angiogenesis by binding to and activating VEGF receptors, such as VEGFR‐1 and VEGFR‐2, on ECs. The VEGF family includes several members: VEGF‐A, ‐B, ‐C, ‐D, ‐E, ‐F, and placental growth factor (PlGF). Among these, VEGF‐A and its isoforms play the major roles in angiogenesis [170]. However, VEGF is costly and possesses a limited biological half‐life. Furthermore, it modulates angiogenesis in a threshold‐dependent manner, potentially leading to adverse dose‐related effects, including undesirable immune responses or cytotoxic effects [171, 172]. A promising alternative to VEGF is bioactive peptide motifs that mimic the growth factor's bioactivity. As a representative example, a synthetic VEGF‐mimetic peptide QK mimicking the 17−25 helix region of VEGF was prepared by D'Andrea et al. [173]. By structurally mimicking this receptor‐binding domain, QK engages and activates VEGFR2, triggering downstream signaling pathways, including FAK, ERK1/2, and AKT1, which are critical for EC migration, proliferation, and permeability [115]. The QK peptide has been incorporated into biomaterials in many studies to promote vascularization and enhance PNR. For example, QK peptide‐encapsulated nanoliposome (QK‐NL) was loaded into an injectable hydrogel to enable sustained peptide release, thereby creating a supportive microenvironment for nerve regeneration (Figure 7A). The QK‐NL significantly enhanced the proliferation, migration, and tube formation of human umbilical vein endothelial cells (HUVECs) via the VEGF signaling pathway in vitro. In a rat model of peripheral nerve crush injury, the QK‐incorporated hydrogel was directly injected into the injury site, resulting in markedly increased micro‐vessel and mature vessel densities at four weeks post‐surgery (Figure 7B,C). Moreover, the increased SFI, the higher wet weight ratio of the gastrocnemius muscle, and the reduced Col fiber area collectively indicated enhanced functional recovery (Figure 7D−G) [115]. In addition, the QK peptide was utilized synergistically with other functional peptides, such as the BDNF mimic peptide [44, 45, 47, 48].

In addition to VEGF, basic fibroblast growth factor (bFGF or FGF2) is known for its pro‐angiogenic properties. FGF2 is a member of the FGF family, which comprises a large group of growth factors that modulate several biological functions, including angiogenesis, tissue granulation, and epidermal regeneration, by binding to FGF receptors (FGFRs) [174]. FGF2 exerts an angiogenic effect by stimulating EC migration and invasion as well as plasminogen activator production [170]. However, the therapeutic efficacy of exogenous recombinant FGFs in vivo is constrained by their high molecular weight, limited diffusion, and immunogenic properties. Therefore, researchers have developed a small peptide, CH02, to target FGFR2. Surface plasmon resonance (SPR) and liquid chromatography‐mass spectrometry (LC‐MS/MS) analysis demonstrated that CH02 directly binds to and activates FGFR2 and VEGFR2. This receptor activation triggers downstream MAPK/ERK and PI3K‐AKT signaling cascades, which are key signaling pathways governing endothelial angiogenic responses, thereby facilitating angiogenesis [175, 176]. In addition, the CH02 peptide exerts effects on neurons and SCs, which further facilitate PNR. The administration of CH02 significantly improves axon regeneration and sensory−motor behavioral recovery following dorsal root crush injury in rats [175].

## 6 Conclusions and Perspectives

Peptides function as potent bioactive factors that markedly enhance the performance of biomaterials in PNR through diverse mechanisms, including promoting axon elongation, reinforcing SC support, modulating inflammatory microenvironments, and inducing vascularization. Notably, most of these peptides are designed to mimic specific protein functions while overcoming associated limitations, such as instability, poor bioavailability, or immunogenicity. These bioactive peptides can be incorporated into biomaterials to promote PNR. Peptides regulate diverse biological processes in PNR, providing multiple opportunities to develop tailored, multifunctional biomaterials.

### 6.1 Challenges and Considerations for Clinical Translation

A deeper mechanistic understanding is required to guide the rational development of peptide‐incorporated biomaterials. First, the impact of peptide incorporation strategies on the release kinetics, spatial distribution, and in vivo degradation remains insufficiently understood. Incorporation into biomaterials, particularly via chemical bonding, may alter peptide structure or conformation, potentially affecting their biological metabolism. For example, PEGylation of peptides not only provides steric hindrance that blocks proteases from accessing cleavage sites but also masks peptides from immune surveillance, reducing immune‐mediated degradation. Whether other biomaterials possess similar properties remains an important area for future investigation and clinical translation. Second, the bioactive efficacy of peptides can be influenced by the biomaterials and conjugation strategies, and this influence may vary under different conditions. For example, Álvarez et al. recently designed a series of peptide amphiphiles integrated with functional peptides, the IKVAV and FGF2 mimetic peptide, and demonstrated that the formed supramolecular scaffolds with greater motion led to improved functional recovery in a murine spinal cord injury model [177]. Such findings highlight the need for further in‐depth or systematic studies to clarify how biomaterials and conjugation strategies modulate peptide bioactivity and therapeutic outcomes.

Another major challenge for clinical translation lies in the limitations of current preclinical models. Most studies on peptide‐incorporated biomaterials for treating PNI have been conducted using rat models, likely because of the relatively recent development of protein‐mimetic peptides and the discovery of new functions in natural peptides. However, the rat model has limitations, including its small size and species‐specific neurobiological regenerative characteristics [178]. Consequently, the biological effects and dynamics observed in rat models may not consistently translate to humans. The use of larger and more clinically relevant animal models will therefore be essential for advancing peptide‐based strategies toward clinical application.

Besides, clinical practicality and manufacturability represent critical but often underappreciated considerations. Although peptides generally exhibit good biocompatibility, combining them with overly complex or controversial biomaterials may impede clinical translation. The use of clinically approved biomaterials, such as PLA and PGA, in combination with peptides may provide an optimal starting point for clinical translation. In another aspect, incorporating peptides into biomaterials may increase storage challenges and reduce clinical practicality compared with pure biomaterials, such as poly(L‑lactide‑*co*‑ε‑caprolactone) (PLCL) conduits. Peptides are biological macromolecules with defined three‐dimensional structures, and their chemical and conformational stability is substantially lower than that of synthetic polymers, such as PLCL. Lyophilized powder is the most common form for long‐term storage of peptide‐based drugs. Therefore, advancing peptide‐incorporated biomaterials into clinical practice requires attention to clinical usability, and a promising approach is to develop these biomaterials in solid form for long‐term storage that can be reconstituted before administration.

### 6.2 Distinctive Advantages and Promising Directions for Clinical Translation

Peptide‐incorporated biomaterials hold considerable promise for improving the precision of PNR. While current studies have primarily focused on increasing the number of regenerating nerve fibers, future research should place greater emphasis on the accuracy of nerve fiber regeneration. Regenerated nerves often fail to reach their original target cells and instead reinnervate target organs at random [179]. This misdirection may partly account for the limited motor and sensory recovery observed after PNI. In this context, peptide‐incorporated biomaterials offer distinct advantages due to their targeting specificity. For example, the C3 peptide selectively promotes motor axon regeneration [95], while calcitonin gene‐related peptide (CGRP) plays a critical role in sensory axon regeneration [180].

Greater attention should be directed toward the role of autonomic nerve fibers in future studies. In addition to motor and sensory fibers, autonomic fibers represent an essential component of peripheral nerves [181]. Sympathetic signaling generated by autonomic fibers is critical for muscle metabolism and neuromuscular junction health. Yet recent studies indicate that sympathetic axon regeneration and the reinnervation of skin and muscle are often deficient. Moreover, growth‐promoting interventions, such as electrical stimulation and bioluminescent optogenetics, have proven ineffective in improving these outcomes. This deficiency has been implicated as a contributing factor to long‐term abnormalities in target muscle energy metabolism and atrophy [182]. Considering their diverse bioactive functions, including effects on autonomic fibers, peptide‐incorporated biomaterials may provide promising strategies. For instance, the ARA290 peptide has been shown to facilitate the repair of autonomic nerve fibers in a mouse model of diabetic autonomic neuropathy [183, 184].

Peptide‐incorporated biomaterials also hold considerable potential for the development of personalized treatments for PNI. Peptides possess diverse and specific bioactive functions. For example, as noted earlier, the IKVAV, YIGSR, and RGD peptides, although all cell adhesion peptides, target different cell types and exert different effects, demonstrating their selectivity. Such specificity will facilitate the future design of personalized therapies, including peptide‐incorporated biomaterials tailored for elderly patients, individuals with chronic injuries, or those with PNI associated with underlying conditions, such as diabetes and autoimmune diseases.

Moreover, bioactive peptides can be readily incorporated into biomaterials in combination, enabling the coordinated integration of multiple biofunctions within a single construct. Such combinatorial peptide strategies are well‐suited to address the complex and multi‐stage nature of PNR by simultaneously targeting distinct biological processes. Indeed, the feasibility and potential benefits of peptide combinations have already been demonstrated in several studies, including the concurrent incorporation of neurotrophic and angiogenic mimetic peptides (e.g., RGI and QK), which has resulted in synergistic regenerative outcomes [45, 47]. Despite these advantages, the rational design and clinical translation of combinatorial peptide‐based biomaterials require careful consideration. Effective combinations should be guided by a sufficiently deep understanding of individual peptides’ mechanisms of action, systematic optimization of peptide composition and ratios, and consideration of patient‐specific factors, including age, comorbidities, and the type of nerve injury. In particular, to accommodate patient‐specific variability, modular or component‐based strategies may offer a flexible and practical approach to PNR by enabling peptides to be combined or adjusted as needed.

## Author Contributions

Z.Z. performed the conceptualization, wrote, reviewed, and edited the final version. J.F.L. wrote, reviewed, and edited the final version. J.C.L. and D.L. reviewed and edited the final version. J.D. performed conceptualization, supervision, reviewed, and edited the final draft. B.L. performed the conceptualization, supervision, and project administration, reviewed and edited the final draft, and acquired the funding acquisition.

## Funding

This work was financially supported by the National Natural Science Foundation of China (Grant No. U23A20490) and the Laboratory Independent Innovation Capacity Building Project of Jilin Provincial Development and Reform Commission (Grant No. 2021C006).

## Conflicts of Interest

The authors declare no conflicts of interest.

## Data Availability

The authors have nothing to report.
